# Synthesis and Activity Evaluation of Novel Benzoxazepinone Derivatives as Potential Inhibitors of Glycogen Phosphorylase

**DOI:** 10.3390/molecules30214249

**Published:** 2025-10-31

**Authors:** Dezhi Peng, Youde Wang, Zhiwei Yan, Shuai Li, Yachun Guo, Tienan Wang, Liying Zhang

**Affiliations:** 1 Laboratory of Traditional Chinese Medicine Research and Development of Hebei Province, Institute of Traditional Chinese Medicine, Chengde Medical University, Chengde 067000, China; 17796935424@163.com (D.P.); wangyoude8686@126.com (Y.W.); cdyanzhiwei@163.com (Z.Y.); cmuyhls@163.com (S.L.); cdwangtienan@163.com (T.W.); 2 Department of Pathogen Biology, Chengde Medical University, Chengde 067000, China; gyc123263295@126.com

**Keywords:** benzoxazepinone derivatives, glycogen phosphorylase inhibitors, biological activity, molecular docking, glycogen degradation

## Abstract

Glycogen phosphorylase (GP) is a key enzyme of glycogen catabolism, so it is significant to discover a new GP inhibitor. A series of benzoxazepinone derivatives were identified as glycogen phosphorylase (GP) inhibitors with potent activity. These compounds exhibited strong inhibitory effects. Among them, compound **8g** (IC_50_ = 0.62 ± 0.16 μM) showed significant inhibitory activity against rabbit muscle glycogen phosphorylase a (GPa). Its inhibition of glycogen degradation in HL-7702 cells was three times greater than that of **PSN-357**. Molecular docking studies revealed that the binding conformation of compound **8g** with PYGL allowed the benzoxazinone moiety to form stable hydrogen-bond networks and hydrophobic interactions, which may explain its excellent activity.

## 1. Introduction

Type 2 diabetes mellitus (T2DM) is a common chronic disease associated with life-threatening complications, which may result in reduced quality of life, increased mortality, and elevated healthcare costs [1,2,3,4,5]. Multiple oral hypoglycemic agents, such as sulfonylureas, biguanides, and thiazolidinediones, are widely prescribed as symptomatic treatments for T2DM. However, these agents are often associated with adverse effects [6,7]. Therefore, the development of novel antidiabetic agents is of critical importance. The latest research indicates that structural derivatives of benzoxazinones may have a positive impact on the treatment of diabetes [8,9,10]. However, the specific inhibitory activities and mechanisms of action of these compounds still need to be further explored, which provides new ideas for the development of novel glycoprotein inhibitors based on this core structure.

Studies have confirmed that abnormal glycogen metabolism is one of the key causes of elevated blood glucose levels. Glycogen phosphorylase (GP) is a key enzyme that catalyzes the breakdown of glycogen to generate glucose for energy production. Studies have demonstrated that inhibiting glycogen phosphorylase activity significantly reduces fasting blood glucose concentrations [11,12,13,14]. Consequently, pharmacological inhibition of GP is considered a promising therapeutic strategy for disorders associated with abnormal glycogen metabolism [15]. GP is an allosterically regulated protein with multiple ligand-binding sites, including the catalytic site, purine nucleoside site, allosteric sites, and the indole site [15,16]. Inhibitors can modulate enzymatic activity by targeting these sites, owing to their crucial roles in glycogen metabolism [17,18,19,20].

**Figure 1:**
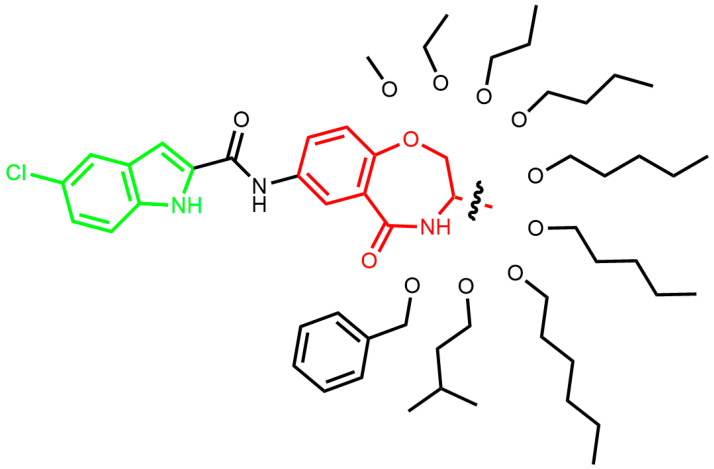
The structure of the target compound.

Our group previously reported that benzoxazepinone derivatives act as GP inhibitors with strong inhibitory activity [7,21]. To elucidate the structure–activity relationship (SAR), we strategically incorporated nonpolar alkyl moieties into the molecular scaffold and synthesized a series of novel indole–oxazepinone-based glycogen phosphorylase inhibitors. Preliminary SAR analysis and GP inhibitory activity evaluation were subsequently conducted (Figure 1).

## 2. Results and Discussion

### 2.1. Chemistry

**Scheme 1:**
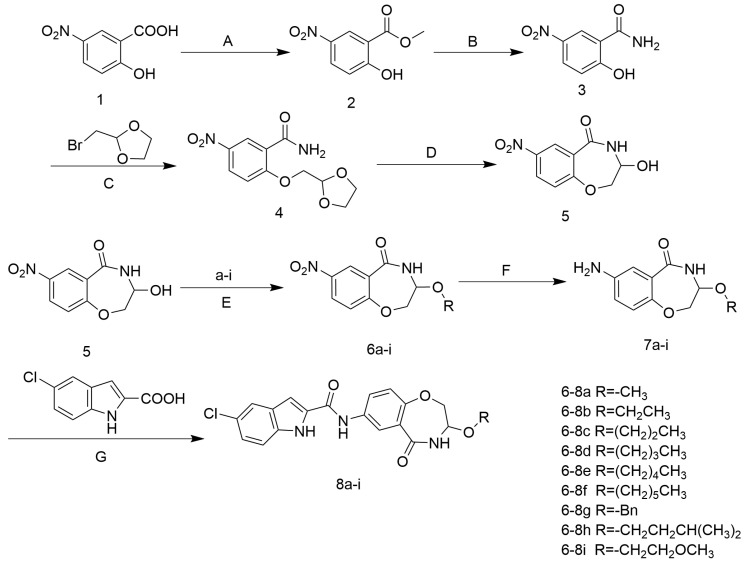
Synthesis routes of benzoxazepinone derivatives. Reagents and conditions: (A) H_2_SO_4_, reflux, CH_3_OH; (B) NH_3_·H_2_O; (C) K_2_CO_3_, KI, 120 °C; (D) 1,4-Dioxane, 1N H_2_SO_4_, 98 °C. (E) TSOH, 50 °C; Note: Compounds **a**–**i** refer to the following esters: methyl butyrate, ethyl acetate, propyl propanoate, butyl butyrate, amyl acetate, hexyl hexanoate, benzyl acetate, isoamyl alcohol, and ethylene glycol methyl ether; (F) HCOONH_4_, Pd/C, 50 °C; (G) HATU, Et_3_N, 45 °C.

In this study, a series of novel glycogen phosphorylase inhibitors were designed and synthesized (Scheme 1). The key intermediate compound **5** was synthesized using 5-nitrosalicylic acid as the starting material. This compound underwent esterification with methanol in the presence of sulfuric acid to yield compound **2** [21]. Under a nitrogen atmosphere, compound **2** was converted to compound **3** via ammonolysis with aqueous ammonia. Subsequently, a nucleophilic substitution reaction with 2-(bromomethyl)-1,3-dioxolane in the presence of potassium carbonate (K_2_CO_3_) and catalytic potassium iodide afforded compound **4**. Finally, hydrolysis and cyclization mediated by sulfuric acid in 1,4-dioxane yielded compound **5**.

In the presence of P-toluenesulfonic Acid, the hydroxyl group at the 3-position of the benzoxazepinone moiety in compound **5** underwent a nucleophilic substitution reaction with ester compounds such as Ethyl Acetate, Propyl Propanoate, and Butyl Butanoate to generate the corresponding ether derivatives **6a**–**i**. Compounds **6a**–**i** were then reduced to the corresponding amines **7a**–**i** using palladium on carbon (Pd/C) and ammonium formate. The amines subsequently underwent amidation with 5-chloroindole-2-carboxylic acid, mediated by the coupling agent HATU and the base triethylamine, affording the target compounds **8a**–**i**.

### 2.2. Phosphorylase Enzyme Assay and SAR Analysis

**Figure 2:**
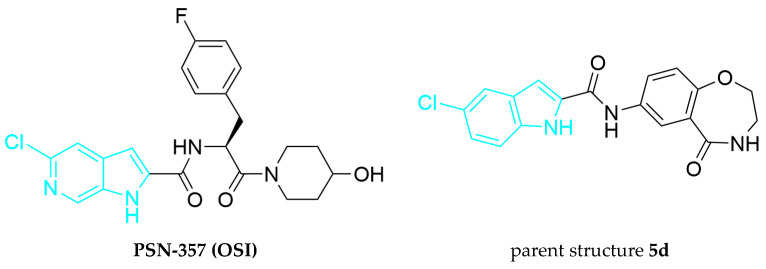
The structure of the positive control.

The synthesized compounds were evaluated for their inhibitory activity against rabbit muscle glycogen phosphorylase a (RMGPa) according to a previously reported protocol [22]. **PSN-357**, a compound in phase II clinical trials, was used as a positive control [23,24]. We also used the parent structure **5d** as a positive control [21] (Figure 2).

This study investigated the effects of introducing aliphatic and aromatic groups via an ether linkage on benzoxazepinone derivatives. Variations in alkyl chain length (homologation), branching, and cyclic structures profoundly influenced the inhibitory activity of the synthesized compounds against RMGPa. As shown in Table 1, most compounds exhibited good activity. Among them, compound **8a** was the most potent, with an IC_50_ value of 0.21 ± 0.05 μM. Its inhibitory activity was twice as strong as that of the positive control **PSN-357** (IC_50_ = 0.42 ± 0.01 μM). And it was more potent than the parent structure **5d** (IC50 = 0.25 ± 0.05 μM). In addition, compounds **8d**, **8e**, **8f**, **8g**, and **8h** also demonstrated significant biological effects.

Preliminary SAR analysis indicated that inhibitory activity decreased with increasing linear alkyl chain length, as observed in the comparison of **8a** with **8b** and **8c**. However, the marked difference in potency between **8f** and **8e**/**8d** suggests that inhibitory activity may increase substantially once the alkyl chain reaches a certain length. The reason for this phenomenon may be that the longer carbon chain of **8f** might en-hance the hydrophobic interaction with the binding pocket of the target, while the chain lengths of **8d**/**8e** might be insufficient to fully occupy the hydrophobic region, resulting in a reduced binding efficiency. The underlying mechanism of this phenomenon remains unclear. The introduction of a benzyl or isopentyl group enhanced inhibitory activity, whereas the introduction of a 2-methoxyethyl group (compound **8i**) resulted in poor activity. Further investigations are required to elucidate the mechanistic details.

### 2.3. Glycogenolysis Inhibition Assay in Liver Cells

To evaluate the effects of the synthesized compounds on glycogen degradation, compounds **8a**–**8i** were subjected to a glycogenolysis assay in HL-7702 cells. The results are summarized in Table 2. Interestingly, their inhibitory activities displayed trends similar to those observed in the GP inhibition assay. Most compounds exhibited good inhibitory activity.

Introduction of alkyl groups into the heterocyclic scaffold led to a reduction in inhibitory activity with increasing linear alkyl chain length (e.g., **8a** vs. **8e**). However, when the chain length reached a certain threshold, a significant increase in activity was observed (e.g., **8e** vs. **8f**/**8h**). The introduction of a benzyl group yielded particularly favorable results, with compound **8g** exhibiting a threefold increase in potency compared with **PSN-357** in HL-7702 cells. Moreover, its potency is twice that of parent structure **5d**. These findings suggest that introducing alkyl or benzyl groups markedly influences inhibitory activity. By contrast, incorporation of a 2-methoxyethyl group resulted in unsatisfactory activity, indicating that such substituents may be detrimental to inhibitory potency. This observed decline in activity could be attributed to the introduction of the additional ether bond, which reduced the overall molecular lipophilicity, thereby compromising cellular permeability.

### 2.4. Binding Mode of Compound ***8g***

**Figure 3:**
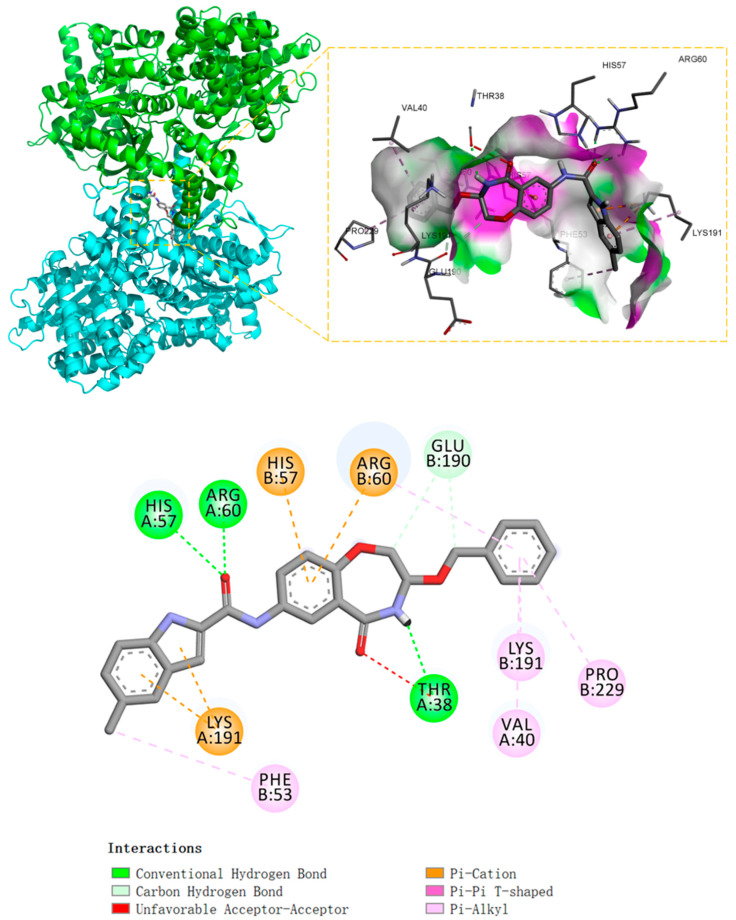
The binding mode of compound **8g** at the active site of human liver glycogen phosphorylase A (PDB ID: 1EXV).

To investigate the molecular mechanism of action, the most potent compound, **8g**, was docked into the binding site of human liver GPa (Protein Data Bank identifier: 1EXV) using Discovery Studio 4.5. Since the commercially available ingliforib shows strong inhibitory activity against liver GP (PYGL), we analyzed the binding mode of **8g** with Enliforib as a reference [5]. Ingliforib exhibits rich receptor-ligand interactions. The docking scores of **8g** with PYGL was −9.83 kcl/mol. As illustrated in Figure 3, within PYGL, the lactam N–H group of **8g** formed a hydrogen bond with the oxygen atom of residue THR38 of chain A (N–H⋯O=C). In addition, the carbonyl oxygen of the central bridge engaged in two hydrogen bonds with residues HIS57 and ARG60 of chain A. Compound **8g** also participated in a carbon–hydrogen bond interaction with residue GLU190 of chain B. The six-membered benzoxazepinone ring formed π–cation interactions with residues HIS57 and ARG60 of chain B, whereas the indole ring established a similar interaction with residue LYS191 of chain A. The terminal benzene ring of compound **8g** engaged in hydrophobic interactions with residue VAL40 of chain A and residues LYS191, PRO229, and ARG60 of chain B. When comparing the complex of compound **8g** with that of ingliforib, there are both similarities and differences in their binding modes. For instance, the carbonyl oxygen on both compounds formed hydrogen bonds with the residue ARG60 of chain A. Additionally, the carbonyl oxygen on the middle bridge of **8g** also formed a hydrogen bond with the B-chain residue HIS57 of chain B. The tail phenyl ring of **8g** formed hydrophobic interactions with residues such as VAL40, while the phenyl ring of ingliforib formed π-π interactions with HIS57. This might be due to the structural differences between them, which lead to certain changes in their interaction modes with amino acid residues. In summary, the binding mode of **8g** with GPa was mediated by hydrophobic stacking, hydrogen bonding, and π–cation interactions. These findings elucidate the molecular mechanism of enzyme–inhibitor interaction and provide a foundation for further structural optimization.

### 2.5. ADMET Predictions of Compound ***8g***

In the process of drug development, it is crucial to evaluate the ADMET properties of novel compounds. Therefore, we predicted the ADMET parameters of compound **8g** using online computational toolkits such as SwissADME and ADMETlab 2.0 hoping to provide guidance for subsequent experiments. Ingliforib, a potent GP inhibitor that has entered the phase II clinical study, was selected as a reference because its pharmacokinetic properties met the drug requirements. The relevant results are summarized in Table 3. Compared with ingliforib, the water solubility of compound **8g** is slightly lower, which is related to the hydrophobic groups in its molecular structure. The two have particularly similar plasma protein binding rates. Furthermore, **8g** is similar to ingliforib and fully complies with the acute toxicity rule, with a median lethal dose (LD_50_) of 2.31 mol/kg, which is slightly inferior to ingliforib. The HIA and hERG inhibition values of compound **8g** are also very close to those of ingliforib. Overall, based on ADMET prediction, compound **8g** is expected to be an ideal drug.

### 2.6. Conclusions

Based on the previous discovery of benzoxazolinone derivatives as potent GP inhibitors, this study conducted an in-depth exploration of such structures. We designed and synthesized a series of novel benzoxazolinone derivatives and systematically evaluated them. Through GP inhibition experiments and structure–activity relationship analysis, we found that most compounds exhibited good GP inhibitory activity, among which compound **8g** (IC50 = 1.51 ± 0.21 μM) showed particularly outstanding inhibitory potential, significantly outperforming other analogues (such as **PSN357** and the parent compound **5d**). Molecular docking studies further revealed that compound **8g** exerts its inhibitory effect by forming stable interactions with key residues in the GP active site (such as H-bonds with ARG60 of chain A and HIS57 of chain B). Additionally, its pharmacokinetic properties were predicted using online computational tools, and the data indicated similarities to ingliforib (such as LD50 and HIA), suggesting that this compound has potential for further development.

This study through a combination of in vitro experiments and computational simulations, systematically elucidated the potential of benzoxazepinone derivatives as GP inhibitors in regulating glycogenolysis, achieving our initial goal of discovering novel and highly active GP inhibitors and preliminarily clarifying their structure–activity relationship and mechanism of action.

However, this study also has certain limitations. All experimental data are based on in vitro models and computational simulation analyses and thus cannot fully reflect the real behavior of such compounds in complex organisms, including their systemic pharmacodynamics characteristics, metabolic stability, and potential toxicity. Therefore, future research should focus on conducting in vivo pharmacodynamics verification in relevant animal models (such as type 2 diabetes rodent models) and systematically investigate their pharmacokinetic/toxicological (ADMET) properties.

Based on the current research results, we suggest that subsequent work can be carried out in the following directions: First, conduct structural optimization on compound **8g** to further enhance its activity and selectivity; second, carry out in vivo pharmacological experiments to clarify its hypoglycemic effect in animal models; third, combine more precise computational simulation and experimental methods to deeply explore the mechanism of action of this type of inhibitor with GP. Currently, our laboratory has conducted in-depth research in the above directions, and the related results will be released successively.

## 3. Materials and Methods

### 3.1. Materials and General Methods

All reagents, solvents were purchased from J&K Chemicals (Beijing, China) and WuXi AppTec (Wuxi, Jiangsu, China). Nuclear magnetic resonance (NMR) spectra were recorded on a Bruker Avance III 400 MHz and a Bruker Fourier 300 MHz spectrometer (Bruker Corporation, Karlsruhe, Germany). Chemical shifts were referenced internally to residual solvent signals relative to TMS (δ = 0.00 ppm). Positive and negative ion LC–MS data were acquired at 303 K using a quadrupole mass spectrometer on an Agilent LC/MSD 1200 series equipped with a 50 × 4.6 mm (5 μm) ODS column (Agilent Technologies, Inc., Santa Clara, CA, USA). High-resolution mass spectrometry (HRMS) was performed on an Agilent 6540 QTOF system. All final compounds were purified to >95% purity, as confirmed by HPLC analysis on an Agilent 1260 instrument (Agilent Technologies, Inc., Singapore) equipped with an Agilent Poroshell 120 EC-C18 column (4.6 × 150 mm, 4.0 μm). Preparative HPLC was carried out using a Flash Welchrom C18 column (150 × 20 mm) (Bonna-Agela Technologies, Tianjin, China). Melting points were determined using an RY-1 melting point meter. Experimental data for intermediate compounds are provided in the Supplementary Materials.

### 3.2. General Procedure for Compound Synthesis

#### 3.2.1. General Procedure for the Synthesis of Compounds **2**–**5**

Methyl 2-hydroxy-5-nitrobenzoate (**2**)

A solution of 5-nitrosalicylic acid (4.0 g, 21.84 mmol) in methanol (70 mL) was acidified by dropwise addition of concentrated sulfuric acid (1.5 mL) with stirring. The reaction mixture was placed under a nitrogen atmosphere and refluxed for 48 h. Upon completion, methanol was removed under reduced pressure. Potassium carbonate was added to the residue until gas evolution ceased. Water (20 mL) was added, and the mixture was extracted with ethyl acetate (3 × 50 mL). The combined organic layers were dried over anhydrous sodium sulfate for 2–3 h, filtered, and concentrated under vacuum. Purification by silica gel column chromatography (petroleum ether/ethyl acetate, 15–20%) afforded the product as a white solid (4.0 g, 93%). HPLC analysis: 100%. m.p. 112–114 °C. ESI-MS *m*/*z*: 239.2 (M+H)^+^. 1H NMR (400 MHz, CDCl3) δ 11.43 (s, 1H), 8.79 (d, *J* = 2.8 Hz, 1H), 8.34 (dd, *J* = 9.2, 2.8 Hz, 1H), 7.09 (d, *J* = 9.2 Hz, 1H), 4.04 (s, 3H). 13C-NMR (100 MHz, CDCl3): 169.3, 166.2, 140.0, 130.6, 126.7, 118.7, 112.1, 53.1.

2-Hydroxy-5-nitrobenzamide (**3**)

Methyl 5-nitrosalicylate (4.0 g, 20.29 mmol) was dissolved in ammonia solution (70 mL) and stirred at 50 °C for 48 h under a nitrogen atmosphere. After completion, the solvent was removed under reduced pressure. The residue was diluted with water (30 mL), carefully acidified to near neutrality with 2 M hydrochloric acid, and extracted with ethyl acetate (3 × 50 mL). The combined organic layers were dried over anhydrous sodium sulfate, filtered, and concentrated. Purification by silica gel column chromatography (petroleum ether/ethyl acetate, 30–50%) yielded the product as a pale yellow solid (2.70 g, 73%). HPLC analysis: 100%. m.p. 130–132 °C. ESI-MS *m*/*z*: 180.7 (M-H)^−^. 1H-NMR (400 MHz, *d*_6_-DMSO): 7.10 (d, *J* = 8.0 Hz, 1H), 8.29 (dd, *J* = 8.0, 4.0 Hz, 2H), 8.81 (s, 1H), 8.90 (d, *J* = 2.4 Hz, 1H), 14.16 (s, 1H). 13C-NMR (100 MHz, d6-DMSO): 170.6, 166.8, 139.5, 129.6, 125.5, 119.0, 115.1.

2-[(1,3-Dioxolan-2-yl)methoxy]-5-nitrobenzamide (**4**)

A solution of 2-hydroxy-5-nitrobenzamide (0.30 g, 1.65 mmol) in DMF (3.0 mL) was treated with potassium carbonate (0.40 g, 2.89 mmol) and stirred at room temperature for 10 min. Subsequently, 2-(bromomethyl)-1,3-dioxolane (0.27 mL, 2.61 mmol) and a catalytic amount of potassium iodide were added. The reaction was carried out under nitrogen at 120 °C for 72 h. The mixture was diluted with brine (20 mL) and extracted with ethyl acetate (3 × 50 mL). The combined organic extracts were washed with saturated brine (3 × 20 mL), dried over anhydrous sodium sulfate, filtered, and concentrated under reduced pressure. Purification by silica gel column chromatography (petroleum ether/ethyl acetate, 35–60%) provided the product as a pale yellow solid (0.22 g, 50%). HPLC analysis: 98%. m.p. 152–154 °C. ESI-MS *m*/*z*: 269.1 (M+H)^+^. ^1^H-NMR (400 MHz, CDCl_3_): 3.99–4.09 (m, 4H), 4.31 (d, *J* = 3.6 Hz, 2H), 5.35 (t, *J* = 3.6 Hz, 1H), 6.08 (s, 1H), 7.10 (d, *J* = 9.2 Hz, 1H), 7.78 (s, 1H), 8.35 (dd, *J* = 9.2, 2.8 Hz, 1H), 9.10 (d, *J* = 3.2 Hz, 1H). ^13^C-NMR (100 MHz, CDCl_3_): 164.4, 160.9, 142.3, 128.9, 128.4, 122.4, 113.0, 100.8, 69.3, 65.6.

7-Nitro-3-hydroxy-3,4-dihydrobenzo[*f*][1,4]oxazepin-5(2*H*)-one (**5**)

2-[(1,3-Dioxolan-2-yl)methoxy]-5-nitrobenzamide (1.5 g, 5.59 mmol) was dissolved in a mixture of 1,4-dioxane (10 mL) and water (30 mL). Concentrated sulfuric acid (1.5 mL) was added dropwise with stirring. The reaction mixture was heated to 98 °C and maintained for 8 h. After cooling, the mixture was extracted with ethyl acetate (3 × 50 mL). The combined organic layers were washed with saturated brine (3 × 20 mL), dried over anhydrous sodium sulfate, filtered, and concentrated under reduced pressure. Purification by silica gel column chromatography (dichloromethane/methanol, 1%) yielded the product as a pale yellow solid (0.48 g, 38%). HPLC analysis: 100%. m.p. 163–165 °C. ESI-MS *m*/*z*: 222.7 (M-H)^−^. ^1^H-NMR (400 MHz, *d*_6_-DMSO): 4.34 (d, *J* = 12.4 Hz, 1H), 4.53 (dd, *J* = 12.4, 4.4 Hz, 1H), 4.93 (q, *J* = 8.8 Hz, 1H), 6.33 (d, *J* = 4.8 Hz, 1H), 7.24 (d, *J* = 9.2 Hz, 1H), 8.26 (dd, *J* = 9.2, 3.2 Hz, 1H), 8.96 (d, *J* = 3.2 Hz, 1H), 9.22 (d, *J* = 5.6 Hz, 1H). ^13^C-NMR (100 MHz, *d*_6_-DMSO): 163.8, 162.1, 141.0, 129.6, 127.5, 121.7, 120.7, 73.7, 72.1.

#### 3.2.2. General Procedure for the Synthesis of Compound **6a**–**i**

7-Nitro-3-hydroxy-3,4-dihydrobenzo[*f*][1,4]oxazepin-5(2*H*)-one (1.0 equiv) was added to a solution containing an excess of esters a–i. p-Toluenesulfonic acid (0.1 equiv) was introduced with continuous stirring. The reaction mixture was maintained at 50 °C for 3 h, cooled to room temperature, and concentrated. The crude product was purified by silica gel column chromatography.

3-Methoxy-7-nitro-3,4-dihydrobenzo[*f*][1,4]oxazepin-5(2*H*)-one (**6a**)

HPLC analysis: 100%. m.p. 195–197 °C. ESI-MS *m*/*z*: 239.2 (M+H)^+^. ^1^H-NMR (400 MHz, CDCl_3_): 3.46 (s, 3H), 4.35 (d, *J* = 12.3 Hz, 1H), 4.68 (d, *J* = 4.8 Hz, 1H), 4.74 (dd, *J* = 12.3, 3.8 Hz, 1H), 7.18 (d, *J* = 9.0 Hz, 1H), 7.72 (s, 1H), 8.26 (dd, *J* = 9.4, 1.8 Hz, 1H), 9.19 (s, 1H). ^13^C-NMR (100 MHz, CDCl_3_): 165.7, 161.7, 142.4, 130.6, 128.3, 121.8, 119.9, 81.0, 72.0, 55.7.

3-ethoxy-7-nitro-3,4-dihydrobenzo[*f*][1,4]oxazepin-5(2*H*)-one (**6b**)

HPLC analysis: 96%. m.p. 149–151 °C. ESI-MS *m*/*z*: 239.2 (M+H)^+^. ^1^H-NMR (400 MHz, CDCl_3_): 1.21 (t, *J* = 7.2 Hz, 3H), 3.57–3.64 (m, 1H), 3.76–3.83 (m, 1H), 4.36 (d, *J* = 12.4 Hz, 1H), 4.72 (dd, *J* = 12.4, 4.0 Hz, 1H), 4.82 (t, *J* = 4.0 Hz, 1H), 7.18 (d, *J* = 9.2 Hz, 1H), 8.26 (dd, *J* = 8.8, 2.0 Hz, 1H), 8.34 (s, 1H), 9.18 (d, *J* = 2.0 Hz, 1H). ^13^C-NMR (100 MHz, CDCl_3_): 166.0, 162.0, 142.3, 130.5, 128.3, 121.9, 120.1, 79.5, 72.5, 63.9, 14.9.

7-nitro-3-propoxy-3,4-dihydrobenzo[*f*][1,4]oxazepin-5(2*H*)-one (**6c**)

HPLC analysis: 93%. m.p. 130–132 °C. ESI-MS *m*/*z*: 289.2 (M+Na)^+^. ^1^H-NMR (400 MHz, CDCl_3_): 0.82 (t, *J* = 7.4 Hz, 3H), 1.53–1.62 (m, 2H), 3.51 (dt, *J* = 9.1, 6.4 Hz, 1H), 3.66 (dt, *J* = 9.1, 6.8 Hz, 1H), 4.38 (d, *J* = 12.2 Hz, 1H), 4.72 (dd, *J* = 12.3, 4.3 Hz, 1H), 4.81 (t, *J* = 4.8 Hz, 1H), 7.18 (d, *J* = 9.1 Hz, 1H), 8.26 (dd, *J* = 9.1, 2.9 Hz, 1H), 8.40 (d, *J* = 5.2 Hz, 1H), 9.17 (d, *J* = 2.9 Hz, 1H). ^13^C-NMR (100 MHz, CDCl_3_): 166.1, 162.0, 142.2, 130.4, 128.2, 121.8, 120.2, 79.6, 77.4, 77.0, 76.7, 72.6, 69.9, 22.6, 10.4.

3-butoxy-7-nitro-3,4-dihydrobenzo[*f*][1,4]oxazepin-5(2*H*)-one (**6d**)

HPLC analysis: 96%. m.p. 122–124 °C. ESI-MS *m*/*z*: 281.5 (M+H)^+^. ^1^H-NMR (500 MHz, CDCl_3_): 0.84 (t, *J* = 7.4 Hz, 3H), 1.23–1.28 (m, 2H), 1.51–1.56 (m, 2H), 3.53 (dt, *J* = 9.2, 6.4 Hz, 1H), 3.70 (dt, *J* = 9.1, 6.8 Hz, 1H), 4.37 (d, *J* = 12.2 Hz, 1H), 4.69 (dd, *J* = 12.3, 4.4 Hz, 1H), 4.79 (t, *J* = 4.8 Hz, 1H), 7.16 (d, *J* = 9.1 Hz, 1H), 8.21 (d, *J* = 4.7 Hz, 1H), 8.25 (dd, *J* = 9.1, 2.9 Hz, 1H), 9.15 (d, *J* = 2.9 Hz, 1H). ^13^C-NMR (125 MHz, CDCl_3_): 166.0, 161.9, 142.3, 130.3, 128.1, 121.8, 120.3, 79.7, 72.6, 68.0, 31.3, 19.0, 13.6.

7-nitro-3-(pentyloxy)-3,4-dihydrobenzo[*f*][1,4]oxazepin-5(2*H*)-one (**6e**)

HPLC analysis: 99%. m.p. 106–108 °C. ESI-MS *m*/*z*: 317.4 (M+Na)^+^. ^1^H-NMR (500 MHz, CDCl_3_): 0.80 (t, *J* = 7.0 Hz, 3H), 1.15–1.27 (m, 4H), 1.52–1.58 (m, 2H), 3.53 (dt, *J* = 9.1, 6.4 Hz, 1H), 3.69 (dt, *J* = 8.8, 6.9 Hz, 1H), 4.38 (d, *J* = 12.3 Hz, 1H), 4.70 (dd, *J* = 12.3, 4.3 Hz, 1H), 4.80 (t, *J* = 4.9 Hz, 1H), 7.17 (d, *J* = 9.1 Hz, 1H), 8.25 (dd, *J* = 9.1, 2.9 Hz, 1H), 8.42 (d, *J* = 4.1 Hz, 1H), 9.14 (d, *J* = 2.8 Hz, 1H). ^13^C-NMR (125 MHz, CDCl_3_): 166.2, 161.9, 142.3, 130.2, 128.1, 121.8, 120.4, 79.6, 72.7, 68.3, 28.9, 28.0, 22.2, 13.8.

3-(hexyloxy)-7-nitro-3,4-dihydrobenzo[*f*][1,4]oxazepin-5(2*H*)-one (**6f**)

HPLC analysis: 99%. m.p. 93–95 °C. ESI-MS *m*/*z*: 331.4 (M+Na)^+^. ^1^H-NMR (500 MHz, CDCl_3_): 0.81 (t, *J* = 6.7 Hz, 3H), 1.16–1.26 (m, 2H), 1.53 (dd, *J* = 13.2, 6.5 Hz, 2H), 3.52 (dt, *J* = 9.1, 6.4 Hz, 1H), 3.69 (dt, *J* = 9.0, 6.8 Hz, 1H), 4.38 (d, *J* = 12.3 Hz, 1H), 4.69 (dd, *J* = 12.3, 4.4 Hz, 1H), 4.79 (t, *J* = 4.9 Hz, 1H), 7.16 (d, *J* = 9.1 Hz, 1H), 8.20 (d, *J* = 3.7 Hz, 1H), 8.25 (dd, *J* = 9.1, 2.9 Hz, 1H), 9.15 (d, *J* = 2.9 Hz, 1H). ^13^C-NMR (125 MHz, CDCl_3_): 166.0, 161.9, 142.3, 130.2, 128.1, 121.7, 120.4, 79.6, 72.6, 68.3, 31.3, 29.2, 25.5, 22.5, 13.8.

3-(benzyloxy)-7-nitro-3,4-dihydrobenzo[*f*][1,4]oxazepin-5(2*H*)-one (**6g**)

HPLC analysis: 100%. m.p. 171–173 °C. ESI-MS *m*/*z*: 337.2 (M+Na)^+^. ^1^H-NMR (500 MHz, CDCl_3_): 4.35 (d, *J* = 12.4 Hz, 1H), 4.63–4.70 (m, 2H), 4.80 (dd, *J* = 14.9, 8.8 Hz, 2H), 7.19 (d, *J* = 9.0 Hz, 1H), 7.32–7.39 (m, 5H), 8.26 (dd, *J* = 9.0, 2.7 Hz, 1H), 8.68 (s, 1H), 9.21 (d, *J* = 2.6 Hz, 1H). ^13^C-NMR (125 MHz, CDCl_3_): 166.4, 162.0, 142.3, 136.2, 130.5, 128.8, 128.4, 128.3, 128.2, 121.9, 120.0, 77.6, 72.5, 69.4.

3-(isopentyloxy)-7-nitro-3,4-dihydrobenzo[*f*][1,4]oxazepin-5(2*H*)-one (**6h**)

HPLC analysis: 100%. m.p. 126–128 °C. ESI-MS *m*/*z*: 295.2 (M+H)^+^. ^1^H-NMR (500 MHz, CDCl_3_): 0.82 (dd, *J* = 9.3, 6.6 Hz, 6H), 1.45 (tt, *J* = 13.7, 6.7 Hz, 2H), 1.55 (dt, *J* = 13.4, 6.7 Hz, 1H), 3.56 (dt, *J* = 9.2, 6.6 Hz, 1H), 3.74 (dt, *J* = 9.2, 6.9 Hz, 1H), 4.37 (d, *J* = 12.2 Hz, 1H), 4.70 (dd, *J* = 12.3, 4.3 Hz, 1H), 4.80 (t, *J* = 3.6 Hz, 1H), 7.16 (d, *J* = 9.1 Hz, 1H), 8.25 (dd, *J* = 9.1, 2.9 Hz, 1H), 8.42 (d, *J* = 4.9 Hz, 1H), 9.15 (d, *J* = 2.9 Hz, 1H). ^13^C-NMR (125 MHz, CDCl_3_): 166.1, 162.0, 142.3, 130.3, 128.1, 121.8, 120.3, 79.6, 72.6, 66.8, 38.0, 24.9, 22.5, 22.2.

3-(2-methoxyethoxy)-7-nitro-3,4-dihydrobenzo[*f*][1,4]oxazepin-5(2*H*)-one (**6i**)

HPLC analysis: 94%. m.p. 115–117 °C. ESI-MS *m*/*z*: 305.1 (M+Na)^+^. ^1^H-NMR (500 MHz, CDCl_3_): 3.37 (s, 3H), 3.55–3.56 (m, 2H), 3.78–3.87 (m, 2H), 4.36 (d, *J* = 12.4 Hz, 1H), 4.70 (dd, *J* = 12.4, 4.5 Hz, 1H), 4.95 (t, *J* = 5.0 Hz, 1H), 7.16 (d, *J* = 9.0 Hz, 1H), 8.12 (d, *J* = 4.3 Hz, 1H), 8.24 (dd, *J* = 9.0, 2.9 Hz, 1H), 9.18 (d, *J* = 2.9 Hz, 1H). ^13^C-NMR (125 MHz, CDCl_3_): 165.3, 162.1, 142.4, 130.5, 128.1, 121.7, 120.4, 80.3, 72.41, 72.38, 67.7, 59.0.

#### 3.2.3. General Procedure for the Synthesis of Compounds **7a**–**i**

Compound **6a**–**i** (1.0 eqv) was dissolved in tetrahydrofuran, followed by the addition of HCOONH_4_ (10.0 eqv) and Pd/C (0.5 eqv). The reaction was carried out with stirring at 50 °C until completion. The reaction mixture was cooled to room temperature and concentrated, and the product was purified by silica gel column chromatography.

7-amino-3-methoxy-3,4-dihydrobenzo[*f*][1,4]oxazepin-5(2*H*)-one (**7a**)

HPLC analysis: 92.6%. ESI-MS *m*/*z*: 209.1 (M+H)^+^. ^1^H-NMR (400 MHz, *d*_6_-DMSO): 3.17 (s, 3H), 4.15 (d, *J* = 3.2 Hz, 2H), 4.46 (dt, *J* = 6.2, 3.2 Hz, 1H), 4.93 (s, 2H), 6.65 (dd, *J* = 8.5, 2.8 Hz, 1H), 6.72 (d, *J* = 8.5 Hz, 1H), 7.05 (d, *J* = 2.8 Hz, 1H), 8.77 (d, *J* = 5.9 Hz, 1H). ^13^C-NMR (100 MHz, *d*_6_-DMSO): 167.8, 147.6, 143.9, 124.9, 120.5, 119.0, 115.1, 81.8, 73.8, 54.5.

7-amino-3-ethoxy-3,4-dihydrobenzo[*f*][1,4]oxazepin-5(2*H*)-one (**7b**)

HPLC analysis: 100%. ESI-MS *m*/*z*: 221.6 (M-H)^−^. ^1^H-NMR (400 MHz, *d*_6_-DMSO): 1.02 (t, *J* = 6.8 Hz, 3H), 3.36–3.42 (m, 1H), 3.51–3.59 (m, 1H), 4.14 (t, *J* = 2.8 Hz, 2H), 4.58 (dt, *J* = 5.6, 3.2 Hz, 1H), 4.92 (s, 2H), 6.66 (dd, *J* = 8.4, 2.8 Hz, 1H), 6.73 (d, *J* = 8.4 Hz, 1H), 7.07 (d, *J* = 2.8 Hz, 1H), 8.72 (d, *J* = 5.6 Hz, 1H). ^13^C-NMR (100 MHz, *d*_6_-DMSO): 168.2, 148.3, 144.4, 125.3, 121.0, 119.5, 115.7, 80.7, 74.4, 62.7,15.4.

7-amino-3-propoxy-3,4-dihydrobenzo[*f*][1,4]oxazepin-5(2*H*)-one (**7c**)

HPLC analysis: 92.6%. ESI-MS *m*/*z*: 209.1 (M+H)^+^. ^1^H-NMR (400 MHz, *d*_6_-DMSO): 0.73 (t, *J* = 7.4 Hz, 3H), 1.35–1.43 (m, 2H), 3.28 (dt, *J* = 9.4, 6.4 Hz, 1H), 3.39–3.44 (m, 1H), 4.15 (d, *J* = 3.1 Hz, 2H), 4.55 (dt, *J* = 5.9, 3.1 Hz, 1H), 4.94 (s, 2H), 6.64 (dd, *J* = 8.5, 2.8 Hz, 1H), 6.72 (d, *J* = 8.5 Hz, 1H), 7.04 (d, *J* = 2.7 Hz, 1H), 8.73 (d, *J* = 5.8 Hz, 1H). ^13^C-NMR (100 MHz, *d*_6_-DMSO): 167.9, 147.7, 143.8, 125.2, 120.5, 118.9, 115.1, 80.3, 74.2, 68.4, 22.2, 10.4.

7-amino-3-butoxy-3,4-dihydrobenzo[*f*][1,4]oxazepin-5(2*H*)-one (**7d**)

HPLC analysis: 97%. ESI-MS *m*/*z*: 251.1 (M+H)^+^. ^1^H-NMR (500 MHz, *d*_6_-DMSO): 0.80 (t, *J* = 7.4 Hz, 3H), 1.13–1.21 (m, 2H), 1.34–1.39 (m, 2H), 3.30–3.33 (m, 1H), 3.48 (dt, *J* = 9.4, 6.7 Hz, 1H), 4.14 (d, *J* = 3.2 Hz, 2H), 4.55 (dt, *J* = 6.2, 3.2 Hz, 1H), 4.87 (s, 2H), 6.65 (dd, *J* = 8.5, 2.8 Hz, 1H), 6.72 (d, *J* = 8.5 Hz, 1H), 7.05 (d, *J* = 2.8 Hz, 1H), 8.66 (d, *J* = 5.8 Hz, 1H). ^13^C-NMR (125 MHz, *d*_6_-DMSO): 167.8, 147.7, 143.8, 125.1, 120.5, 118.9, 115.2, 80.4, 74.2, 66.4, 30.9, 18.6, 13.5.

7-amino-3-(pentyloxy)-3,4-dihydrobenzo[*f*][1,4]oxazepin-5(2*H*)-one (**7e**)

HPLC analysis: 100%. ESI-MS *m*/*z*: 265.2 (M+H)^+^. ^1^H-NMR (400 MHz, *d*_6_-DMSO): 0.81 (t, *J* = 6.8 Hz, 3H), 1.08–1.16 (m, 2H), 1.17–1.26 (m, 2H), 1.33–1.42 (m, 2H), 3.30 (q, *J* = 7.2 Hz, 1H), 3.46 (q, *J* = 8.4 Hz, 1H), 4.14 (s, 2H), 4.54 (d, *J* = 2.8 Hz, 1H), 4.89 (s, 1H), 6.65 (d, *J* = 8.4 Hz, 1H), 6.72 (d, *J* = 8.8 Hz, 1H), 7.04 (s, 1H), 8.69 (d, *J* = 5.6 Hz, 1H). ^13^C-NMR (100 MHz, *d*_6_-DMSO): 167.9, 147.7, 143.9, 125.2, 120.5, 118.8, 115.1, 80.4, 74.2, 66.7, 28.6, 27.7, 21.8, 13.9.

7-amino-3-(hexyloxy)-3,4-dihydrobenzo[*f*][1,4]oxazepin-5(2*H*)-one (**7f**)

HPLC analysis: 99%. ESI-MS *m*/*z*: 279.1 (M+H)^+^. ^1^H-NMR (500 MHz, CDCl_3_): 0.86 (t, *J* = 6.9 Hz, 3H), 1.23–1.28 (m, 6H), 1.57–1.48 (m, 2H), 3.44 (dt, *J* = 9.1, 6.5 Hz, 1H), 3.65 (dt, *J* = 9.0, 6.8 Hz, 1H), 4.22 (dd, *J* = 12.0, 2.9 Hz, 1H), 4.31 (dd, *J* = 12.0, 4.6 Hz, 1H), 4.69 (td, *J* = 4.8, 3.3 Hz, 1H), 6.77 (dd, *J* = 8.6, 2.9 Hz, 1H), 6.88 (d, *J* = 8.6 Hz, 1H), 7.09 (d, *J* = 3.9 Hz, 1H), 7.36 (d, *J* = 2.9 Hz, 1H). ^13^C-NMR (125 MHz, CDCl_3_): 167.9, 150.4, 141.8, 123.8, 121.5, 121.0, 117.2, 81.8, 73.8, 68.5, 31.5, 29.4, 25.6, 22.5, 13.9.

7-amino-3-(benzyloxy)-3,4-dihydrobenzo[*f*][1,4]oxazepin-5(2*H*)-one (**7g**)

HPLC analysis: 95%. ESI-MS *m*/*z*: 285.2 (M+H)^+^. ^1^H-NMR (500 MHz, *d*_6_-DMSO): 4.17–4.23 (m, 2H), 4.42 (d, *J* = 12.0 Hz, 1H), 4.56 (d, *J* = 12.0 Hz, 1H), 4.68–4.69 (m, 1H), 4.91 (s, 2H), 6.66 (dd, *J* = 8.5, 2.7 Hz, 1H), 6.74 (d, *J* = 8.5 Hz, 1H), 7.08 (d, *J* = 2.6 Hz, 1H), 7.20 (d, *J* = 7.2 Hz, 2H), 7.27 (dq, *J* = 14.3, 7.2 Hz, 3H), 8.82 (d, *J* = 5.5 Hz, 1H). ^13^C-NMR (125 MHz, *d*_6_-DMSO): 168.4, 148.3, 144.4, 138.3, 128.6, 128.0, 127.9, 125.5, 121.0, 119.5, 115.7, 80.5, 74.5, 68.8.

7-amino-3-(isopentyloxy)-3,4-dihydrobenzo[*f*][1,4]oxazepin-5(2*H*)-one (**7h**)

HPLC analysis: 92.6%. ESI-MS *m*/*z*: 265.2 (M+H)^+^. ^1^H-NMR (500 MHz, *d*_6_-DMSO): 0.78 (d, *J* = 6.7 Hz, 6H), 1.20–1.32 (m, 2H), 1.42–1.51 (m, 1H), 3.30–3.35 (m, 1H), 3.52 (dt, *J* = 9.5, 6.9 Hz, 1H), 4.14 (d, *J* = 3.2 Hz, 2H), 4.89 (s, 2H), 4.52–4.56 (m, 1H), 6.65 (dd, *J* = 8.5, 2.8 Hz, 1H), 6.72 (dd, *J* = 8.5, 2.3 Hz, 1H), 7.05 (d, *J* = 2.8 Hz, 1H), 8.67 (d, *J* = 5.9 Hz, 1H). ^13^C-NMR (125 MHz, *d*_6_-DMSO): 168.3, 148.2, 144.3, 125.6, 121.0, 119.4, 115.7, 80.9, 74.6, 65.6, 38.3, 24.8, 23.0, 22.6.

7-amino-3-(2-methoxyethoxy)-3,4-dihydrobenzo[*f*][1,4]oxazepin-5(2*H*)-one (**7i**)

HPLC analysis: 92.6%. ESI-MS *m*/*z*: 275.1 (M+Na)^+^. ^1^H-NMR (500 MHz, *d*_6_-DMSO): 3.17 (s, 3H), 3.42–3.34 (m, 2H), 3.53–3.45 (m, 1H), 3.60 (ddd, *J* = 10.6, 5.5, 4.1 Hz, 1H), 4.15 (qd, *J* = 12.3, 3.2 Hz, 2H), 4.61 (dt, *J* = 6.1, 3.2 Hz, 1H), 4.89 (s, 2H), 6.66 (dd, *J* = 8.5, 2.8 Hz, 1H), 6.73 (d, *J* = 8.5 Hz, 1H), 7.09 (d, *J* = 2.7 Hz, 1H), 8.67 (d, *J* = 5.5 Hz, 1H). ^13^C-NMR (125 MHz, *d*_6_-DMSO): 168.0, 148.4, 144.3, 125.1, 121.0, 119.6, 115.8, 81.3, 74.2, 71.3, 66.7, 58.4.

#### 3.2.4. General Procedure for the Synthesis of Compounds **8a**–**i**

A solution of 5-chloroindole-2-carboxylic acid (1.0 eqv) in DMF was sequentially treated with HATU (1.0 eqv in DMF) and N,N-diisopropylethylamine (DIPEA, 3.0 eqv in DMF). After stirring at room temperature for 10 min, compounds **7a**–**i** (1.0 eqv in DMF) were added. The reaction was heated to 45 °C and stirred for 5 h. Upon completion, the mixture was cooled to room temperature, and 50 mL of purified water was added, followed by suction filtration. The product was purified by silica gel column chromatography.

5-chloro-*N*-(3-methoxy-5-oxo-2,3,4,5-tetrahydrobenzo[*f*][1,4]oxazepin-7-yl)-1*H*-indole-2-carboxamide (**8a**)

HPLC analysis: 100%. m.p. None. ESI-MS *m*/*z*: 420.8 (M+Cl)^−^. ^1^H-NMR (400 MHz, *d*_6_-DMSO): 3.24 (s, 3H), 4.24 (d, *J* = 12.2 Hz, 1H), 4.45 (d, *J* = 12.3 Hz, 1H), 4.55 (s, 1H), 7.05 (d, *J* = 8.7 Hz, 1H), 7.23 (d, *J* = 8.2 Hz, 1H), 7.43 (s, 1H), 7.48 (d, *J* = 8.6 Hz, 1H), 7.78 (s, 1H), 7.98 (d, *J* = 7.1 Hz, 1H), 8.33 (s, 1H), 9.06 (d, *J* = 5.2 Hz, 1H), 10.39 (s, 1H), 11.94 (s, 1H). ^13^C-NMR (100 MHz, *d*_6_-DMSO): 166.5, 159.1, 153.1, 135.1, 133.0, 132.9, 128.1, 125.6, 124.3, 123.8, 123.6, 122.2, 120.8, 120.3, 113.9, 103.2, 80.8, 72.4, 54.5.

5-chloro-*N*-(3-ethoxy-5-oxo-2,3,4,5-tetrahydrobenzo[*f*][1,4]oxazepin-7-yl)-1*H*-indole-2-carboxamide (**8b**)

HPLC analysis: 100%. m.p. 227–229 °C. ESI-MS *m*/*z*: 435.8 (M+Cl)^−^. ^1^H-NMR (400 MHz, *d*_6_-DMSO): 1.05 (t, *J* = 7.0 Hz, 3H), 3.44 (dq, *J* = 9.6 Hz, 7.2 Hz, 1H), 3.60 (dq, *J* = 9.6 Hz, 7.2 Hz, 1H), 4.22 (dd, *J* = 12.4, 1.6 Hz, 1H), 4.43 (dd, *J* = 12.4, 3.6 Hz, 1H), 4.65–4.67 (m, 1H), 7.05 (d, *J* = 8.8 Hz, 1H), 7.23 (dd, *J* = 8.4, 2.0 Hz, 1H), 7.43 (d, *J* = 1.2 Hz, 1H), 7.48 (d, *J* = 8.8 Hz, 1H), 7.78 (d, *J* = 2.0 Hz, 1H), 7.98 (dd, *J* = 8.8, 2.4 Hz, 1H), 8.33 (d, *J* = 2.8 Hz, 1H), 9.04 (d, *J* = 6.0 Hz, 1H), 10.39 (s, 1H), 11.94 (s, 1H). ^13^C-NMR (100 MHz, *d*_6_-DMSO): 166.4, 159.1, 153.2, 135.1, 132.92, 132.86, 128.1, 125.6, 124.3, 123.8, 123.7, 122.1, 120.8, 120.3, 113.9, 103.2, 79.2, 72.6, 62.2, 14.9.

5-chloro-*N*-(5-oxo-3-propoxy-2,3,4,5-tetrahydrobenzo[*f*][1,4]oxazepin-7-yl)-1*H*-indole-2-carboxamide (**8c**)

HPLC analysis: 100%. m.p. 231–233 °C. ESI-MS *m*/*z*: 449.8 (M+Na)^+^. ^1^H-NMR (400 MHz, *d*_6_-DMSO): 0.74 (t, *J* = 7.4 Hz, 3H), 1.39–1.48 (m, 2H), 3.34–3.39 (m, 1H), 3.45–3.51 (m, 1H), 4.25 (d, *J* = 12.0 Hz, 1H), 4.43 (dd, *J* = 12.3, 3.3 Hz, 1H), 4.60–4.69 (m, 1H), 7.06 (d, *J* = 8.8 Hz, 1H), 7.23 (dd, *J* = 8.7, 1.8 Hz, 1H), 7.43 (s, 1H), 7.48 (d, *J* = 8.7 Hz, 1H), 7.78 (d, *J* = 1.2 Hz, 1H), 7.98 (dd, *J* = 8.8, 2.6 Hz, 1H), 8.32 (d, *J* = 2.5 Hz, 1H), 9.03 (d, *J* = 5.9 Hz, 1H), 10.38 (s, 1H), 11.94 (s, 1H). ^13^C-NMR (100 MHz, *d*_6_-DMSO): 166.6, 159.1, 153.1, 135.1, 133.0, 132.9, 128.1, 125.5, 124.3, 123.8, 123.6, 122.5, 120.8, 120.3, 113.9, 103.2, 79.4, 72.8, 68.3, 22.2, 10.4.

*N*-(3-butoxy-5-oxo-2,3,4,5-tetrahydrobenzo[*f*][1,4]oxazepin-7-yl)-5-chloro-1*H*-indole-2-carboxamide (**8d**)

HPLC analysis: 100%. m.p. 224–226 °C. ESI-MS *m*/*z*: 463.8 (M+Cl)^−^. ^1^H-NMR (500 MHz, *d*_6_-DMSO): 0.80 (t, *J* = 7.4 Hz, 3H), 1.16–1.19 (m, 2H), 1.38–1.42 (m, 2H), 3.40 (dt, *J* = 9.6, 6.3 Hz, 1H), 3.54 (dt, *J* = 9.4, 6.8 Hz, 1H), 4.24 (dd, *J* = 12.3, 1.6 Hz, 1H), 4.42 (dd, *J* = 12.3, 3.4 Hz, 1H), 4.61–4.66 (m, 1H), 7.04 (d, *J* = 8.8 Hz, 1H), 7.22 (dd, *J* = 8.7, 1.9 Hz, 1H), 7.42 (d, *J* = 1.1 Hz, 1H), 7.48 (d, *J* = 8.7 Hz, 1H), 7.76 (d, *J* = 1.3 Hz, 1H), 7.96 (dd, *J* = 8.8, 2.7 Hz, 1H), 8.31 (d, *J* = 2.6 Hz, 1H), 8.96 (d, *J* = 6.0 Hz, 1H), 10.34 (s, 1H), 11.88 (s, 1H). ^13^C-NMR (125 MHz, *d*_6_-DMSO): 167.1, 159.6, 153.6, 135.6, 133.5, 133.4, 128.6, 125.9, 124.9, 124.3, 124.1, 123.1, 121.2, 120.8, 114.4, 103.7, 80.0, 73.4, 66.8, 31.4, 19.1, 14.0.

5-chloro-*N*-(5-oxo-3-(pentyloxy)-2,3,4,5-tetrahydrobenzo[*f*][1,4]oxazepin-7-yl)-1*H*-indole-2-carboxamide (**8e**)

HPLC analysis: 100%. m.p. 222–224 °C. ESI-MS *m*/*z*: 477.9 (M+Cl)^−^. ^1^H-NMR (400 MHz, *d*_6_-DMSO): 0.78 (t, *J* = 6.4 Hz, 3H), 1.09–1.14 (m, 2H), 1.16–1.19 (m, 2H), 1.40–1.44 (m, 2H), 3.38 (q, *J* = 6.0 Hz, 1H), 3.51 (q, *J* = 8.0 Hz, 1H), 4.25 (d, *J* = 12.4 Hz, 1H), 4.42 (d, *J* = 11.6 Hz, 1H), 4.64 (d, *J* = 2.0 Hz, 1H), 7.05 (d, *J* = 8.8 Hz, 1H), 7.23 (d, *J* = 8.8 Hz, 1H), 7.43 (s, 1H), 7.48 (d, *J* = 8.4 Hz, 1H), 7.77 (s, 1H), 7.98 (d, *J* = 8.8 Hz, 1H), 8.31 (s, 1H), 8.99 (d, *J* = 5.6 Hz, 1H), 10.36 (s, 1H), 11.91 (s, 1H). ^13^C-NMR (100 MHz, *d*_6_-DMSO): 166.7, 159.1, 153.1, 135.1, 133.1, 132.9, 128.1, 125.3, 124.4, 123.8, 123.4, 122.9, 120.8, 120.3, 113.9, 103.2, 79.5, 73.1, 66.6, 28.6, 27.7, 21.7, 13.8.

5-chloro-*N*-(3-(hexyloxy)-5-oxo-2,3,4,5-tetrahydrobenzo[*f*][1,4]oxazepin-7-yl)-1*H*-indole-2-carboxamide (**8f**)

HPLC analysis: 100%. m.p. 187–189 °C. ESI-MS *m*/*z*: 490.1 (M+Cl)^−^. ^1^H-NMR (500 MHz, *d*_6_-DMSO): 0.77 (t, *J* = 6.2 Hz, 3H), 1.14 (s, 6H), 1.40 (s, 2H), 3.38 (dd, *J* = 14.6, 5.9 Hz, 1H), 3.51 (dd, *J* = 15.4, 6.5 Hz, 1H), 4.25 (d, *J* = 12.0 Hz, 1H), 4.41 (d, *J* = 10.3 Hz, 1H), 4.63 (s, 1H), 7.04 (d, *J* = 8.7 Hz, 1H), 7.22 (d, *J* = 8.5 Hz, 1H), 7.41 (s, 1H), 7.48 (d, *J* = 8.6 Hz, 1H), 7.76 (s, 1H), 7.97 (d, *J* = 8.3 Hz, 1H), 8.30 (s, 1H), 8.95 (d, *J* = 5.5 Hz, 1H), 10.33 (s, 1H), 11.88 (s, 1H). ^13^C-NMR (125 MHz, *d*_6_-DMSO): 167.2, 159.6, 153.5, 135.6, 133.6, 133.4, 128.6, 125.8, 124.8, 124.3, 123.9, 123.4, 121.2, 120.8, 114.4, 103.7, 80.0, 73.6, 67.1, 31.3, 29.3, 25.7, 22.5, 14.3.

*N*-(3-(benzyloxy)-5-oxo-2,3,4,5-tetrahydrobenzo[*f*][1,4]oxazepin-7-yl)-5-chloro-1*H*-indole-2-carboxamide (**8g**)

HPLC analysis: 100%. m.p. 253–255 °C. ESI-MS *m*/*z*: 497.4 (M+Cl)^−^. ^1^H-NMR (500 MHz, *d*_6_-DMSO): 4.29 (dd, *J* = 12.3, 1.4 Hz, 1H), 4.47–4.52 (m, 2H), 4.63 (d, *J* = 12.0 Hz, 1H), 4.77–4.78 (m, 1H), 7.07 (d, *J* = 8.8 Hz, 1H), 7.24 (dt, *J* = 6.5, 3.9 Hz, 4H), 7.28–7.31 (m, 2H), 7.43 (s, 1H), 7.49 (d, *J* = 8.7 Hz, 1H), 7.77 (d, *J* = 1.3 Hz, 1H), 7.98 (dd, *J* = 8.8, 2.6 Hz, 1H), 8.36 (d, *J* = 2.6 Hz, 1H), 9.11 (d, *J* = 6.0 Hz, 1H), 10.36 (s, 1H), 11.90 (s, 1H). ^13^C-NMR (125 MHz, *d*_6_-DMSO): 167.1, 159.6, 153.7, 138.2, 135.6, 133.6, 133.4, 128.7, 128.6, 128.0, 127.9, 126.1, 124.9, 124.3, 124.1, 123.1, 121.2, 120.8, 114.4, 103.8, 79.6, 73.3, 68.8.

5-chloro-*N*-(3-(isopentyloxy)-5-oxo-2,3,4,5-tetrahydrobenzo[*f*][1,4]oxazepin-7-yl)-1*H*-indole-2-carboxamide (**8h**)

HPLC analysis: 100%. m.p. 95–97 °C. ESI-MS *m*/*z*: 477.9 (M+Cl)^−^. ^1^H-NMR (500 MHz, *d*_6_-DMSO): 0.78 (dd, *J* = 6.6, 1.7 Hz, 6H), 1.21–1.37 (m, 2H), 1.45–1.53 (m, 1H), 3.41 (dd, *J* = 15.9, 6.4 Hz, 1H), 3.58 (dd, *J* = 16.4, 7.0 Hz, 1H), 4.24 (d, *J* = 12.1 Hz, 1H), 4.42 (dd, *J* = 12.3, 3.2 Hz, 1H), 4.64 (d, *J* = 2.2 Hz, 1H), 7.04 (d, *J* = 8.8 Hz, 1H), 7.22 (dd, *J* = 8.7, 1.7 Hz, 1H), 7.42 (s, 1H), 7.49 (d, *J* = 8.7 Hz, 1H), 7.76 (s, 1H), 7.96 (dd, *J* = 8.8, 2.3 Hz, 1H), 8.29–8.34 (m, 1H), 8.95 (s, 1H), 10.33 (s, 1H), 11.87 (s, 1H). ^13^C-NMR (125 MHz, *d*_6_-DMSO): 167.1, 159.6, 153.6, 135.6, 133.5, 133.4, 128.6, 125.9, 124.9, 124.3, 124.0, 123.1, 121.2, 120.8, 114.4, 103.7, 80.0, 73.4, 65.5, 38.2, 24.9, 22.9, 22.6.

5-chloro-*N*-[3-(2-methoxyethoxy)-5-oxo-2,3,4,5-tetrahydrobenzo[*f*][1,4]oxazepin-7-yl]-1*H*-indole-2-carboxamide (**8i**)

HPLC analysis: 99%. m.p. 206–208 °C. ESI-MS *m*/*z*: 465.9 (M+Cl)^−^. ^1^H-NMR (500 MHz, *d*_6_-DMSO): 3.18 (s, 3H), 3.39 (tt, *J* = 10.8, 5.6 Hz, 2H), 3.54–3.58 (m, 1H), 3.64–3.69 (m, 1H), 4.23 (d, *J* = 12.4 Hz, 1H), 4.44 (dd, *J* = 12.4, 3.6 Hz, 1H), 4.69–4.70 (m, 1H), 7.05 (d, *J* = 8.8 Hz, 1H), 7.22 (dd, *J* = 8.7, 1.9 Hz, 1H), 7.42 (s, 1H), 7.48 (d, *J* = 8.7 Hz, 1H), 7.76 (s, 1H), 7.98 (dd, *J* = 8.8, 2.7 Hz, 1H), 8.34 (d, *J* = 2.7 Hz, 1H), 8.97 (d, *J* = 5.8 Hz, 1H), 10.35 (s, 1H), 11.89 (s, 1H). ^13^C-NMR (125 MHz, *d*_6_-DMSO): 166.3, 159.1, 153.2, 135.1, 133.0, 132.9, 128.0, 125.5, 124.3, 123.7, 122.1, 120.7, 120.3, 113.9, 103.2, 79.8, 72.4, 70.8, 66.2, 57.9.

### 3.3. In Vitro Bioassays

#### 3.3.1. Enzyme Kinetics

The inhibitory activity of the test compounds against RMGPa was evaluated using a microplate reader (Bio-Rad Laboratories, Berkeley, CA, USA) according to a previously described method [22]. Briefly, RMGPa activity was determined by measuring the release of phosphate from glucose-1-phosphate, which reflects the direction of glycogen synthesis. Each test compound was dissolved in DMSO and diluted to different concentrations for IC_50_ determination. The enzyme was added to 100 μL reaction mixtures in 96-well Costar microplates (Corning Inc., Corning, NY, USA) containing 50 mM HEPES (pH 7.2), 100 mM potassium chloride (KCl), 2.5 mM magnesium chloride (MgCl_2_), 0.5 mM glucose-1-phosphate, 1 mg/mL glycogen, and the test compounds. After incubation at 22 °C for 25 min, 150 μL of a solution containing 10 mg/mL ammonium molybdate and 0.38 mg/mL malachite green was added. The absorbance of phosphate was measured at 620 nm. IC_50_ values were estimated by fitting the inhibition data to a dose–response curve using a logistic regression equation.

#### 3.3.2. Glycogenolysis Inhibition in HL-7702 Cells

The inhibitory effect on hepatic glycogenolysis was quantitatively assessed by colorimetry using a microplate reader (Bio-Rad Laboratories) and anthrone reagent (Sigma-Aldrich, St. Louis, MO, USA), as previously described [22]. Primary human liver HL-7702 cells (National Collection of Authenticated Cell Cultures) were treated with test compounds or DMSO solvent (final concentration, 0.10%) and then co-incubated with 0.3 nM glucagon for 60 min. After termination of the reaction by centrifugation, the cells were digested with 30% potassium hydroxide, and the glycogen content was determined. When culturing cells to concentration, a 6-well plate is used for cell culture, with a cell seeding density of 10^6^ cells per well. The condition is set at a constant temperature of 37 degrees Celsius. The repetition count is three times. The activity assessment criteria were based on the positive drug psn-357 and the parent nuclear compound **5d**. IC_50_ values were estimated by fitting the inhibition data to a logistic regression equation to generate dose–response curves. Analysis was conducted using SPSS Statistics 17.0. In vitro data are presented as mean ± SD, and in vivo data as mean ± SEM. Differences between two groups were assessed using Student’s *t*-test, and comparisons among three or more groups using ANOVA. A *p*-value < 0.05 was considered significant.

### 3.4. Molecular Docking

The molecular structure of compound **8g** was drawn using ChemDraw 20.0 and energy-minimized with the MM2 force field to generate a mol2 file. The three-dimensional structure of glycogen phosphorylase (GP, PDB: 1EXV) was obtained from the Protein Data Bank and used as the receptor protein. Molecular docking between the key protein target GP and compound **8g** was performed using AutoDock Vina 1.1.2. The resulting docking poses were visualized and analyzed with PyMOL and Discovery Studio 4.5. A binding energy of less than −5 kcal/mol for compound **8g** with the target protein indicated a stable interaction.

## Figures and Tables

**Table 1:** RMGPa inhibition assay for conjugates **8a**–**i**.

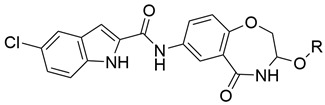		
**Compound**	**R**	**RMGPa** **IC**_**50**_ **± SD (µM)**
**8a**	-CH_3_	0.21 ± 0.05
**8b**	-CH_2_CH_3_	1.61 ± 0.41
**8c**	-(CH_2_)_2_CH_3_	NI
**8d**	-(CH_2_)_3_CH_3_	0.71 ± 0.25
**8e**	-(CH_2_)_4_CH_3_	0.88 ± 0.03
**8f**	-(CH_2_)_5_CH_3_	0.29 ± 0.10
**8g**	-Bn	0.62 ± 0.16
**8h**	-CH_2_CH_2_CH(CH_3_)_2_	0.70 ± 0.55
**8i**	-CH_2_CH_2_OCH_3_	5.31 ± 0.62
**PSN-357**		0.42 ± 0.01
**5d**		0.25 ± 0.05

Each value represents the mean ± standard deviation of three determinations; Using **PSN-357** and **5d** as reference compounds; RMGPa: Rabbit muscle glycogen phosphorylase a.

**Table 2:** IC_50_ values (µM) for glycogenolysis assay results.

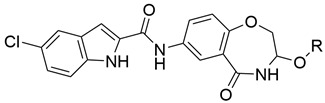		
**Compound**	**R**	**HL-7702** **IC**_**50**_ **± SD (µM)**
**8a**	-CH_3_	8.51 ± 3.99
**8b**	-CH_2_CH_3_	6.77 ± 0.93
**8c**	-(CH_2_)_2_CH_3_	5.33 ± 1.39
**8d**	-(CH_2_)_3_CH_3_	9.81 ± 1.39
**8e**	-(CH_2_)_4_CH_3_	15.38 ± 0.89
**8f**	-(CH_2_)_5_CH_3_	7.67 ± 1.98
**8g**	-Bn	1.51 ± 0.21
**8h**	-CH_2_CH_2_CH(CH_3_)_2_	6.99 ± 0.99
**8i**	-CH_2_CH_2_OCH_3_	NI
**PSN-357**		4.94 ± 0.28
**5d**		3.75 ± 2.67

Each value represents the mean ± standard deviation of three determinations; Using **PSN-357** and **5d** as reference compounds.

**Table 3:** The predicted ADMET through online software.

Compound	Aqueous Solubility ^a^	Plasma Protein Binding ^b^	Acute Oral Toxicity	LD_50_^c^	HIA ^d^	hERG Inhibition ^e^
**8g**	−3.9176	0.983	0 alerts	2.31	0.9423	0.9744
ingliforib	−3.0938	0.973	0 alerts	2.46	0.9743	0.9575

^a^ Unit: logS. ^b^ Unit: 100%. ^c^ Unit: mol/kg. ^d^ Unit: 100%, Human intestinal absorption (HIA). ^e^ in vitro Human ether-a-go-go related gene channel inhibition.

## Data Availability

The data presented in this study are available on request from the corresponding author.

## Supplementary Materials

The following supporting information can be downloaded at: https://www.mdpi.com/article/10.3390/molecules30214249/s1, File S1: Experimental details for intermediate stages; File S2: The mass spectrum of compound **8a**–**i**.
