# Supplementary material for: Synthesis and Activity Evaluation of Novel Benzoxazepinone Derivatives as Potential Inhibitors of Glycogen Phosphorylase
Source: Molecules. 2025 Oct 31;30(21):4249. doi: 10.3390/molecules30214249 (PMC12608647; doi:10.3390/molecules30214249)
Supplement: Supplementary file 1 [file molecules-30-04249-s001.zip › experimental details for intermediate stages.pdf]

3-Methoxy-7-nitro-3,4-dihydrobenzo[*f*][1,4]oxazepin-5(2*H*)-one (6a)

7-Nitro-3-hydroxy-3,4-dihydrobenzo[*f*][1,4]oxazepin-5(2*H*)-one (0.20 g, 0.89 mmol) was added to Methyl butanoate (5 mL). P-Toluenesulfonic acid (10 mg, 0.06 mmol) was subsequently introduced under stirring, and the mixture was heated to 50°C with continuous stirring for 3 hours. After cooling to room temperature, the reaction mixture was concentrated under reduced pressure. Purification by silica gel column chromatography (petroleum ether/ethyl acetate, 15%-50% gradient) afforded the product as a white solid (0.12 g, 57% yield). HPLC analysis: 100%. m.p. 195-197℃.ESI-MS *m/z*: 239.2 (M+H)$^+$. ^{1}H-NMR (400 MHz, $CDCl_3$): 3.46 (s, 3H), 4.35 (d, J = 12.3 Hz, 1H), 4.68 (d, J = 4.8 Hz, 1H), 4.74 (dd, J = 12.3, 3.8 Hz, 1H), 7.18 (d, J = 9.0 Hz, 1H), 7.72 (s, 1H), 8.26 (dd, J = 9.4, 1.8 Hz, 1H), 9.19 (s, 1H). ^{13}C-NMR (100 MHz, $CDCl_3$): 165.7, 161.7, 142.4, 130.6, 128.3, 121.8, 119.9, 81.0, 72.0, 55.7.

7-amino-3-methoxy-3,4-dihydrobenzo[*f*][1,4]oxazepin-5(2*H*)-one (7a)

3-Methoxy-7-nitro-3,4-dihydrobenzo[*f*][1,4]oxazepin-5(2*H*)-one (0.10 g, 0.42 mmol) was dissolved in Tetrahydrofuran (15 mL). To this solution, Ammonium formate (0.30 g, 4.76 mmol) and Palladium on Carbon (15 mg, 0.14 mmol) were added sequentially under stirring. The reaction mixture was then heated to 50°C with continuous stirring for 3 hours. After cooling to room temperature, the mixture was filtered through Celite® and concentrated under reduced pressure. Purification by silica gel column chromatography (dichloromethane/methanol, 0%-2% gradient) yielded the product as a colorless viscous oil (65 mg, 75%). HPLC purity: 92.6%. ESI-MS: m/z 209.1 (M+H)+.^{1}H-NMR (400 MHz, d_6-DMSO): 3.17 (s, 3H), 4.15 (d, J = 3.2 Hz, 2H), 4.46 (dt, J = 6.2, 3.2 Hz, 1H), 4.93 (s, 2H), 6.65 (dd, J = 8.5, 2.8 Hz, 1H), 6.72 (d, J = 8.5 Hz, 1H), 7.05 (d, J = 2.8 Hz, 1H), 8.77 (d, J = 5.9 Hz, 1H). ^{13}C-NMR (100 MHz, d_6-DMSO): 167.8, 147.6, 143.9, 124.9, 120.5, 119.0, 115.1, 81.8, 73.8, 54.5.

5-chloro-*N*-(3-methoxy-5-oxo-2,3,4,5-tetrahydrobenzo[*f*][1,4]oxazepin-7-yl)-1*H*-indole-2-carboxamide (8a)

5-Chloro-1H-indole-2-carboxylic acid (55 mg, 0.28 mmol) was dissolved in N,N-dimethylformamide (1 mL). To this solution, a solution of HATU (106 mg, 0.28 mmol) in N,N-dimethylformamide (1 mL) and a solution of triethylamine (0.14 mL, 0.98 mmol) in N,N-dimethylformamide (0.5 mL) were added sequentially under stirring. After stirring at room temperature for 10 minutes, a solution of 7-amino-3-methoxy-3,4-dihydrobenzo[f][1,4]oxazepin-5(2H)-one (50 mg, 0.24 mmol) in N,N-dimethylformamide (1 mL) was added, and the mixture was heated to 45°C with continuous

stirring for 5 hours. The reaction mixture was cooled to room temperature, quenched with water (30 mL), and filtered. Purification by silica gel column chromatography (dichloromethane/methanol, 0%-2% gradient) afforded the product as a white solid (45 mg, 49%). HPLC analysis: 100%. m.p. 无℃.

ESI-MS *m/z*: 420.8 (M+Cl)$^-$.

^{1}H-NMR (400 MHz, d_6-DMSO): 3.24 (s, 3H), 4.24 (d, J = 12.2 Hz, 1H), 4.45 (d, J = 12.3 Hz, 1H), 4.55 (s, 1H), 7.05 (d, J = 8.7 Hz, 1H), 7.23 (d, J = 8.2 Hz, 1H), 7.43 (s, 1H), 7.48 (d, J = 8.6 Hz, 1H), 7.78 (s, 1H), 7.98 (d, J = 7.1 Hz, 1H), 8.33 (s, 1H), 9.06 (d, J = 5.2 Hz, 1H), 10.39 (s, 1H), 11.94 (s, 1H). ^{13}C-NMR (100 MHz, d_6-DMSO): 166.5, 159.1, 153.1, 135.1, 133.0, 132.9, 128.1, 125.6, 124.3, 123.8, 123.6, 122.2, 120.8, 120.3, 113.9, 103.2, 80.8 , 72.4, 54.5.

3-ethoxy-7-nitro-3,4-dihydrobenzo[*f*][1,4]oxazepin-5(2*H*)-one (6b)

7-Nitro-3-hydroxy-3,4-dihydrobenzo[*f*][1,4]oxazepin-5(2*H*)-one (65 mg, 0.29 mmol) was dissolved in ethyl acetate (5 mL). To this solution, P-toluenesulfonic acid (5 mg, 0.03 mmol) was added under stirring, and the mixture was heated to 50°C with continuous stirring for 3 hours. After cooling to room temperature, the reaction mixture was concentrated under reduced pressure. Purification by silica gel column chromatography (petroleum ether/ethyl acetate, 15%-45% gradient) afforded the product as a white solid (51 mg, 70%). HPLC analysis: 96%. m.p. 149-151℃.

ESI-MS *m/z*: 239.2 (M+H)$^+$.

^{1}H-NMR (400 MHz, $CDCl_3$): 1.21 (t, J = 7.2 Hz, 3H), 3.57-3.64 (m, 1H), 3.76-3.83 (m, 1H), 4.36 (d, J = 12.4 Hz, 1H), 4.72 (dd, J = 12.4, 4.0 Hz, 1H), 4.82 (t, J = 4.0 Hz, 1H), 7.18 (d, J = 9.2 Hz, 1H), 8.26 (dd, J = 8.8, 2.0 Hz, 1H), 8.34 (s, 1H), 9.18 (d, J = 2.0 Hz, 1H). ^{13}C-NMR (100 MHz, $CDCl_3$): 166.0, 162.0, 142.3, 130.5, 128.3, 121.9, 120.1, 79.5, 72.5, 63.9, 14.9.

7-amino-3-ethoxy-3,4-dihydrobenzo[*f*][1,4]oxazepin-5(2*H*)-one (7b)

3-Ethoxy-7-nitro-3,4-dihydrobenzo[*f*][1,4]oxazepin-5(2*H*)-one (50 mg, 0.20 mmol) was dissolved in Tetrahydrofuran (10 mL). To this solution, Ammonium formate (0.20 g, 3.17 mmol) and 10% Palladium on Carbon (10 mg, 0.09 mmol) were added under stirring, and the mixture was heated to 50°C with continuous stirring for 3 hours. After cooling to room temperature, the reaction mixture was filtered through Celite, and the filtrate was concentrated under reduced pressure. Purification by silica gel column chromatography (dichloromethane/methanol, 0%-2% gradient) afforded the product as a colorless viscous oil (30 mg, 68%). HPLC analysis: 100%.

ESI-MS *m/z*: 221.6 (M-H)$^-$.

^{1}H-NMR (400 MHz, d_6-DMSO): 1.02 (t, J = 6.8 Hz, 3H), 3.36-3.42 (m, 1H), 3.51-3.59 (m, 1H), 4.14 (t, J = 2.8 Hz, 2H), 4.58 (dt, J = 5.6, 3.2 Hz, 1H), 4.92 (s, 2H), 6.66 (dd, J = 8.4, 2.8 Hz, 1H), 6.73 (d, J = 8.4 Hz, 1H), 7.07 (d, J = 2.8 Hz, 1H), 8.72 (d, J = 5.6 Hz, 1H). ^{13}C-NMR (100 MHz, d_6-DMSO): 168.2, 148.3, 144.4, 125.3, 121.0, 119.5, 115.7, 80.7, 74.4, 62.7,15.4.

5-chloro-*N*-(3-ethoxy-5-oxo-2,3,4,5-tetrahydrobenzo[*f*][1,4]oxazepin-7-yl)-1*H*-indole-2-carboxamide (8b)

5-Chloro-2-indolecarboxylic acid (50 mg, 0.26 mmol) was dissolved in N,N-dimethylformamide (1 mL). To this solution, a solution of HATU (85 mg, 0.22 mmol) in N,N-dimethylformamide (1 mL) and a solution of Triethylamine (0.13 mL, 0.91 mmol) in N,N-dimethylformamide (0.5 mL) were added sequentially under stirring. The mixture was stirred at room temperature for 10 minutes, followed by the addition of a solution of 7-amino-3-ethoxy-3,4-dihydrobenzo[*f*][1,4]oxazepin-5(2*H*)-one (50 mg, 0.23 mmol) in N,N-dimethylformamide (1 mL). The reaction was then heated to 45°C and stirred for 5 hours. After cooling to room temperature, the reaction mixture was poured into 30 mL of water and filtered. Purification by silica gel column chromatography (dichloromethane/methanol, 0%-2% gradient) afforded the product as a white solid (60 mg, 67%). HPLC analysis: 100%. m.p. 227-229℃.

ESI-MS *m/z*: 435.8 (M+Cl)$^-$.

^{1}H-NMR (400 MHz, d_6-DMSO): 1.05 (t, J = 7.0 Hz, 3H), 3.44 (dq, J = 9.6 Hz, 7.2 Hz, 1H), 3.60 (dq, J = 9.6 Hz, 7.2 Hz, 1H), 4.22 (dd, J = 12.4, 1.6 Hz, 1H), 4.43 (dd, J = 12.4, 3.6 Hz, 1H), 4.65-4.67 (m, 1H), 7.05 (d, J = 8.8 Hz, 1H), 7.23 (dd, J = 8.4, 2.0 Hz, 1H), 7.43 (d, J = 1.2 Hz, 1H), 7.48 (d, J = 8.8 Hz, 1H), 7.78 (d, J = 2.0 Hz, 1H), 7.98 (dd, J = 8.8, 2.4 Hz, 1H), 8.33 (d, J = 2.8 Hz, 1H), 9.04 (d, J = 6.0 Hz, 1H), 10.39 (s, 1H), 11.94 (s, 1H). ^{13}C-NMR (100 MHz, d_6-DMSO): 166.4, 159.1, 153.2, 135.1, 132.92, 132.86, 128.1, 125.6, 124.3, 123.8, 123.7, 122.1, 120.8, 120.3, 113.9, 103.2, 79.2, 72.6, 62.2, 14.9.

7-nitro-3-propoxy-3,4-dihydrobenzo[*f*][1,4]oxazepin-5(2*H*)-one (6c)

7-Nitro-3-hydroxy-3,4-dihydrobenzo[*f*][1,4]oxazepin-5(2*H*)-one (80 mg, 0.36 mmol) was dissolved in Propyl Propanoate (3.5 mL). To this solution, P-toluenesulfonic acid (9 mg, 0.05 mmol) was added under stirring, and the mixture was heated to 50°C with continuous stirring for 10 hours. After cooling to room temperature, the reaction mixture was concentrated under reduced pressure. Purification by silica gel column chromatography (petroleum ether/ethyl acetate, 15%-40% gradient) afforded the

product as a white solid (55 mg, 58%). HPLC analysis: 93%. m.p. 130-132°C.

ESI-MS *m/z*: 289.2 $(M+Na)^+$.

^{1}H-NMR (400 MHz, $CDCl_3$): 0.82 (t, J = 7.4 Hz, 3H), 1.53-1.62 (m, 2H), 3.51 (dt, J = 9.1, 6.4 Hz, 1H), 3.66 (dt, J = 9.1, 6.8 Hz, 1H), 4.38 (d, J = 12.2 Hz, 1H), 4.72 (dd, J = 12.3, 4.3 Hz, 1H), 4.81 (t, J = 4.8 Hz, 1H), 7.18 (d, J = 9.1 Hz, 1H), 8.26 (dd, J = 9.1, 2.9 Hz, 1H), 8.40 (d, J = 5.2 Hz, 1H), 9.17 (d, J = 2.9 Hz, 1H). ^{13}C-NMR (100 MHz, $CDCl_3$): 166.1, 162.0, 142.2, 130.4, 128.2, 121.8, 120.2, 79.6, 77.4, 77.0, 76.7, 72.6, 69.9, 22.6, 10.4.

7-amino-3-propoxy-3,4-dihydrobenzo[*f*][1,4]oxazepin-5(2*H*)-one (7c)

3-Propoxy-7-nitro-3,4-dihydrobenzo[*f*][1,4]oxazepin-5(2*H*)-one (80 mg, 0.30 mmol) was dissolved in Tetrahydrofuran (15 mL). To this solution, Ammonium formate (0.30 g, 4.76 mmol) and 10% Palladium on Carbon (15 mg, 0.14 mmol) were added under stirring, and the mixture was heated to 50°C with continuous stirring for 3 hours. After cooling to room temperature, the reaction mixture was filtered through Celite, and the filtrate was concentrated under reduced pressure. Purification by silica gel column chromatography (dichloromethane/methanol, 0%-2% gradient) afforded the product as a colorless viscous oil (50 mg, 71%). HPLC analysis: 92.6%.

ESI-MS *m/z*: 209.1 $(M+H)^+$.

^{1}H-NMR (400 MHz, d_6-DMSO): 0.73 (t, J = 7.4 Hz, 3H), 1.35-1.43 (m, 2H), 3.28 (dt, J = 9.4, 6.4 Hz, 1H), 3.39-3.44 (m, 1H), 4.15 (d, J = 3.1 Hz, 2H), 4.55 (dt, J = 5.9, 3.1 Hz, 1H), 4.94 (s, 2H), 6.64 (dd, J = 8.5, 2.8 Hz, 1H), 6.72 (d, J = 8.5 Hz, 1H), 7.04 (d, J = 2.7 Hz, 1H), 8.73 (d, J = 5.8 Hz, 1H). ^{13}C-NMR (100 MHz, d_6-DMSO): 167.9, 147.7, 143.8, 125.2, 120.5, 118.9, 115.1, 80.3, 74.2, 68.4, 22.2, 10.4.

5-chloro-*N*-(5-oxo-3-propoxy-2,3,4,5-tetrahydrobenzo[*f*][1,4]oxazepin-7-yl)-1*H*-indole-2-carboxamide (8c)

5-Chloro-2-indolecarboxylic acid (35 mg, 0.18 mmol) was dissolved in N,N-dimethylformamide (1 mL). To this solution, a solution of HATU (65 mg, 0.17 mmol) in N,N-dimethylformamide (1 mL) and a solution of triethylamine (0.08 mL, 0.60 mmol) in N,N-dimethylformamide (0.5 mL) were added sequentially under stirring. The mixture was stirred at room temperature for 10 minutes, followed by the addition of a solution of 7-amino-3-propoxy-3,4-dihydrobenzo[*f*][1,4]oxazepin-5(2*H*)-one (40 mg, 0.17 mmol) in N,N-dimethylformamide (1 mL). The reaction was then heated to 45°C and stirred for 5 hours. After cooling to room temperature, the reaction mixture was poured into 30 mL of water and

filtered. Purification by silica gel column chromatography (dichloromethane/methanol, 0%-2% gradient) afforded the product as a white solid (34 mg, 49%). HPLC analysis: 100%. m.p. 231-233℃.

ESI-MS *m/z*: 449.8 (M+Na)$^+$.

^{1}H-NMR (400 MHz, d_6-DMSO): 0.74 (t, J = 7.4 Hz, 3H), 1.39-1.48 (m, 2H), 3.34-3.39 (m, 1H), 3.45-3.51 (m, 1H), 4.25 (d, J = 12.0 Hz, 1H), 4.43 (dd, J = 12.3, 3.3 Hz, 1H), 4.60-4.69 (m, 1H), 7.06 (d, J = 8.8 Hz, 1H), 7.23 (dd, J = 8.7, 1.8 Hz, 1H), 7.43 (s, 1H), 7.48 (d, J = 8.7 Hz, 1H), 7.78 (d, J = 1.2 Hz, 1H), 7.98 (dd, J = 8.8, 2.6 Hz, 1H), 8.32 (d, J = 2.5 Hz, 1H), 9.03 (d, J = 5.9 Hz, 1H), 10.38 (s, 1H), 11.94 (s, 1H). ^{13}C-NMR (100 MHz, d_6-DMSO): 166.6, 159.1, 153.1, 135.1, 133.0, 132.9, 128.1, 125.5, 124.3, 123.8, 123.6, 122.5, 120.8, 120.3, 113.9, 103.2, 79.4, 72.8, 68.3, 22.2, 10.4.

3-butoxy-7-nitro-3,4-dihydrobenzo[*f*][1,4]oxazepin-5(2*H*)-one (6d)

7-Nitro-3-hydroxy-3,4-dihydrobenzo[*f*][1,4]oxazepin-5(2*H*)-one (0.20 g, 0.89 mmol) was dissolved in N-butyl Butanoate (10 mL). To this solution, P-toluenesulfonic acid (10 mg, 0.06 mmol) was added under stirring, and the mixture was heated to 50°C with continuous stirring for 12 hours. After cooling to room temperature, the reaction mixture was concentrated under reduced pressure. Purification by silica gel column chromatography (petroleum ether/ethyl acetate, 15%-40% gradient) afforded the product as a white solid (185 mg, 74%). HPLC analysis: 96%. m.p. 122-124℃.

ESI-MS *m/z*: 281.5 (M+H)$^+$.

^{1}H-NMR (500 MHz, $CDCl_3$): 0.84 (t, J = 7.4 Hz, 3H), 1.23-1.28 (m, 2H), 1.51-1.56 (m, 2H), 3.53 (dt, J = 9.2, 6.4 Hz, 1H), 3.70 (dt, J = 9.1, 6.8 Hz, 1H), 4.37 (d, J = 12.2 Hz, 1H), 4.69 (dd, J = 12.3, 4.4 Hz, 1H), 4.79 (t, J = 4.8 Hz, 1H), 7.16 (d, J = 9.1 Hz, 1H), 8.21 (d, J = 4.7 Hz, 1H), 8.25 (dd, J = 9.1, 2.9 Hz, 1H), 9.15 (d, J = 2.9 Hz, 1H). ^{13}C-NMR (125 MHz, $CDCl_3$): 166.0, 161.9, 142.3, 130.3, 128.1, 121.8, 120.3, 79.7, 72.6, 68.0, 31.3, 19.0, 13.6.

7-amino-3-butoxy-3,4-dihydrobenzo[*f*][1,4]oxazepin-5(2*H*)-one (7d)

3-Butoxy-7-nitro-3,4-dihydrobenzo[*f*][1,4]oxazepin-5(2*H*)-one (0.16 g, 0.57 mmol) was dissolved in Tetrahydrofuran (5 mL). To this solution, Ammonium formate (0.40 g, 6.35 mmol) and 10% Pd/C (30 mg, 0.28 mmol) were added under stirring, and the mixture was heated to 50°C for 3 hours. After cooling to room temperature, the reaction mixture was filtered through Celite, concentrated under reduced pressure, and purified by silica gel column chromatography (dichloromethane/methanol, 0%-2% gradient) to afford the product as a colorless viscous oil (0.13 g, 90%). HPLC analysis: 97%.

ESI-MS *m/z*: 251.1 (M+H)$^+$.

^{1}H-NMR (500 MHz, d_6-DMSO): 0.80 (t, J = 7.4 Hz, 3H), 1.13-1.21 (m, 2H), 1.34-1.39 (m, 2H), 3.30-3.33 (m, 1H), 3.48 (dt, J = 9.4, 6.7 Hz, 1H), 4.14 (d, J = 3.2 Hz, 2H), 4.55 (dt, J = 6.2, 3.2 Hz, 1H), 4.87 (s, 2H), 6.65 (dd, J = 8.5, 2.8 Hz, 1H), 6.72 (d, J = 8.5 Hz, 1H), 7.05 (d, J = 2.8 Hz, 1H), 8.66 (d, J = 5.8 Hz, 1H). ^{13}C-NMR (125 MHz, d_6-DMSO): 167.8, 147.7, 143.8, 125.1, 120.5, 118.9, 115.2, 80.4, 74.2, 66.4, 30.9, 18.6, 13.5.

***N*-(3-butoxy-5-oxo-2,3,4,5-tetrahydrobenzo[*f*][1,4]oxazepin-7-yl)-5-chloro-1*H*-indole-2-carboxamide (8d)**

5-Chloroindole-2-carboxylic acid (70 mg, 0.36 mmol) was dissolved in N,N-dimethylformamide (1 mL). To this solution, a mixture of HATU (137 mg, 0.36 mmol) in DMF (1 mL) and Triethylamine (0.17 mL, 1.26 mmol) in DMF (0.5 mL) was added dropwise under stirring at room temperature. After stirring for 10 minutes, a solution of 7-amino-3-butoxy-3,4-dihydrobenzo[*f*][1,4]oxazepin-5(2*H*)-one (90 mg, 0.36 mmol) in DMF (1 mL) was added, and the mixture was heated to 45°C for 5 hours. The reaction mixture was cooled to room temperature, diluted with water (30 mL), and filtered. The crude product was purified by silica gel column chromatography (dichloromethane/methanol, 0%-2% gradient) to afford the product as a white solid (85 mg, 55%). HPLC analysis: 100%. m.p. 224-226°C. ESI-MS *m/z*: 463.8 (M+Cl)$^-$.

^{1}H-NMR (500 MHz, d_6-DMSO): 0.80 (t, J = 7.4 Hz, 3H), 1.16-1.19 (m, 2H), 1.38-1.42 (m, 2H), 3.40 (dt, J = 9.6, 6.3 Hz, 1H), 3.54 (dt, J = 9.4, 6.8 Hz, 1H), 4.24 (dd, J = 12.3, 1.6 Hz, 1H), 4.42 (dd, J = 12.3, 3.4 Hz, 1H), 4.61-4.66 (m, 1H), 7.04 (d, J = 8.8 Hz, 1H), 7.22 (dd, J = 8.7, 1.9 Hz, 1H), 7.42 (d, J = 1.1 Hz, 1H), 7.48 (d, J = 8.7 Hz, 1H), 7.76 (d, J = 1.3 Hz, 1H), 7.96 (dd, J = 8.8, 2.7 Hz, 1H), 8.31 (d, J = 2.6 Hz, 1H), 8.96 (d, J = 6.0 Hz, 1H), 10.34 (s, 1H), 11.88 (s, 1H). ^{13}C-NMR (125 MHz, d_6-DMSO): 167.1, 159.6, 153.6, 135.6, 133.5, 133.4, 128.6, 125.9, 124.9, 124.3, 124.1, 123.1, 121.2, 120.8, 114.4, 103.7, 80.0, 73.4, 66.8, 31.4, 19.1, 14.0.

7-nitro-3-(pentyloxy)-3,4-dihydrobenzo[*f*][1,4]oxazepin-5(2*H*)-one (6e)

7-Nitro-3-hydroxy-3,4-dihydrobenzo[f][1,4]oxazepin-5(2H)-one (0.20 g, 0.88 mmol) was dissolved in a mixed solvent of Amyl Acetate (6 mL) and Dioxane (2 mL). To this solution, P-toluenesulfonic acid (20 mg, 0.12 mmol) was added under stirring, and the mixture was heated to 50°C for 15 hours. After cooling to room temperature, the reaction mixture was concentrated under reduced pressure and purified by silica gel column chromatography (petroleum ether/ethyl acetate, 15%-50% gradient) to afford the product as a white solid (0.20 g, 76%). HPLC analysis: 99%. m.p. 106-108°C.

ESI-MS *m/z*: 317.4 (M+Na)$^+$.

^{1}H-NMR (500 MHz, $CDCl_3$): 0.80 (t, J = 7.0 Hz, 3H), 1.15-1.27 (m, 4H), 1.52-1.58 (m, 2H), 3.53 (dt, J = 9.1, 6.4 Hz, 1H), 3.69 (dt, J = 8.8, 6.9 Hz, 1H), 4.38 (d, J = 12.3 Hz, 1H), 4.70 (dd, J = 12.3, 4.3 Hz, 1H), 4.80 (t, J = 4.9 Hz, 1H), 7.17 (d, J = 9.1 Hz, 1H), 8.25 (dd, J = 9.1, 2.9 Hz, 1H), 8.42 (d, J = 4.1 Hz, 1H), 9.14 (d, J = 2.8 Hz, 1H). ^{13}C-NMR (125 MHz, $CDCl_3$): 166.2, 161.9, 142.3, 130.2, 128.1, 121.8, 120.4, 79.6, 72.7, 68.3, 28.9, 28.0, 22.2, 13.8.

7-amino-3-(pentyloxy)-3,4-dihydrobenzo[*f*][1,4]oxazepin-5(2*H*)-one (7e)

3-Pentyloxy-7-nitro-3,4-dihydrobenzo[*f*][1,4]oxazepin-5(2*H*)-one (0.18 g, 0.61 mmol) was dissolved in Tetrahydrofuran (15 mL). To this solution, Ammonium formate (0.40 g, 6.35 mmol) and Palladium on Carbon (15 mg, 0.14 mmol) were added under stirring, and the mixture was heated to 50°C for 4 hours. After cooling to room temperature, the reaction mixture was filtered through Celite and concentrated under reduced pressure. Purification by silica gel column chromatography (dichloromethane/methanol, 0%-2% gradient) afforded the product as a colorless viscous oil (0.15 g, 92%). HPLC analysis: 100%.

ESI-MS *m/z*: 265.2 (M+H)$^+$.

^{1}H-NMR (400 MHz, d_6-DMSO): 0.81 (t, J = 6.8 Hz, 3H), 1.08-1.16 (m, 2H), 1.17-1.26 (m, 2H), 1.33-1.42 (m, 2H), 3.30 (q, J = 7.2 Hz, 1H), 3.46 (q, J = 8.4 Hz, 1H), 4.14 (s, 2H), 4.54 (d, J = 2.8 Hz, 1H), 4.89 (s, 1H), 6.65 (d, J = 8.4 Hz, 1H), 6.72 (d, J = 8.8 Hz, 1H), 7.04 (s, 1H), 8.69 (d, J = 5.6 Hz, 1H). ^{13}C-NMR (100 MHz, d_6-DMSO): 167.9, 147.7, 143.9, 125.2, 120.5, 118.8, 115.1, 80.4, 74.2, 66.7, 28.6, 27.7, 21.8, 13.9.

5-chloro-*N*-(5-oxo-3-(pentyloxy)-2,3,4,5-tetrahydrobenzo[*f*][1,4]oxazepin-7-yl)-1*H*-indole-2-carboxamide (8e)

5-Chloroindole-2-carboxylic acid (103 mg, 0.53 mmol) was dissolved in N,N-dimethylformamide (1.5 mL). A solution of HATU (202 mg, 0.53 mmol) in DMF (1.5 mL) and triethylamine (188 mg, 1.86 mmol) in DMF (0.5 mL) was added sequentially under stirring. After stirring at room temperature for 10 min, a solution of 7-amino-3-pentoxy-3,4-dihydrobenzo[f][1,4]oxazepin-5(2H)-one (140 mg, 0.53 mmol) in DMF (1.5 mL) was added, and the mixture was heated to 45°C with stirring for 5 h. The reaction mixture was cooled to room temperature, quenched with 50 mL of deionized water, and filtered. Purification by silica gel column chromatography (dichloromethane/methanol, 0%-2% gradient) afforded the desired product as a white solid (140 mg, 60%). HPLC analysis: 100%. m.p. 222-224℃.

ESI-MS *m/z*: 477.9 (M+Cl)$^-$.

^{1}H-NMR (400 MHz, d_6-DMSO): 0.78 (t, J = 6.4 Hz, 3H), 1.09-1.14 (m, 2H), 1.16-1.19 (m, 2H), 1.40-1.44 (m, 2H), 3.38 (q, J = 6.0 Hz, 1H), 3.51 (q, J = 8.0 Hz, 1H), 4.25 (d, J = 12.4 Hz, 1H), 4.42 (d, J = 11.6 Hz, 1H), 4.64 (d, J = 2.0 Hz, 1H), 7.05 (d, J = 8.8 Hz, 1H), 7.23 (d, J = 8.8 Hz, 1H), 7.43 (s, 1H), 7.48 (d, J = 8.4 Hz, 1H), 7.77 (s, 1H), 7.98 (d, J = 8.8 Hz, 1H), 8.31 (s, 1H), 8.99 (d, J = 5.6 Hz, 1H), 10.36 (s, 1H), 11.91 (s, 1H). ^{13}C-NMR (100 MHz, d_6-DMSO): 166.7, 159.1, 153.1, 135.1, 133.1, 132.9, 128.1, 125.3, 124.4, 123.8, 123.4, 122.9, 120.8, 120.3, 113.9, 103.2, 79.5, 73.1, 66.6, 28.6, 27.7, 21.7, 13.8.

3-(hexyloxy)-7-nitro-3,4-dihydrobenzo[*f*][1,4]oxazepin-5(2*H*)-one (6f)

7-Nitro-3-hydroxy-3,4-dihydrobenzo[*f*][1,4]oxazepin-5(2*H*)-one (185 mg, 0.83 mmol) was added to a mixed solution of Caproic acid hexyl ester (10 mL) and Dioxane (2 mL). P-Toluenesulfonic acid (20 mg, 0.12 mmol) was then added under stirring, and the mixture was heated to 50°C with stirring for 18 h. After cooling to room temperature, the reaction mixture was concentrated under reduced pressure. Purification by silica gel column chromatography (petroleum ether/ethyl acetate, 20%-60% gradient) afforded the desired product as a white solid (145 mg, 57%). HPLC analysis: 99%. m.p. 93-95℃.

ESI-MS *m/z*: 331.4 (M+Na)$^+$.

^{1}H-NMR (500 MHz, $CDCl_3$): 0.81 (t, J = 6.7 Hz, 3H), 1.16-1.26 (m, 2H), 1.53 (dd, J = 13.2, 6.5 Hz, 2H), 3.52 (dt, J = 9.1, 6.4 Hz, 1H), 3.69 (dt, J = 9.0, 6.8 Hz, 1H), 4.38 (d, J = 12.3 Hz, 1H), 4.69 (dd, J = 12.3, 4.4 Hz, 1H), 4.79 (t, J = 4.9 Hz, 1H), 7.16 (d, J = 9.1 Hz, 1H), 8.20 (d, J = 3.7 Hz, 1H), 8.25 (dd, J = 9.1, 2.9 Hz, 1H), 9.15 (d, J = 2.9 Hz, 1H). ^{13}C-NMR (125 MHz, $CDCl_3$): 166.0, 161.9, 142.3, 130.2, 128.1, 121.7, 120.4, 79.6, 72.6, 68.3, 31.3, 29.2, 25.5, 22.5, 13.8.

7-amino-3-(hexyloxy)-3,4-dihydrobenzo[*f*][1,4]oxazepin-5(2*H*)-one (7f)

3-Hexyloxy-7-nitro-3,4-dihydrobenzo[*f*][1,4]oxazepin-5(2*H*)-one (0.12 g, 0.39 mmol) was dissolved in THF (5 mL), followed by addition of Ammonium formate (0.40 g, 6.35 mmol) and Palladium on Carbon (15 mg, 0.14 mmol). The mixture was stirred at 50°C until complete consumption of starting material (monitored by TLC). After cooling to room temperature, the reaction mixture was filtered through Celite and concentrated under reduced pressure. Purification by silica gel column chromatography (dichloromethane/methanol, 0%-2% gradient) afforded the desired product as a brown viscous oil (96 mg, 89%). HPLC analysis: 99%.

ESI-MS *m/z*: 279.1 (M+H)$^+$.

^{1}H-NMR (500 MHz, $CDCl_3$): 0.86 (t, J = 6.9 Hz, 3H), 1.23-1.28 (m, 6H), 1.57-1.48 (m, 2H), 3.44 (dt, J = 9.1, 6.5 Hz, 1H), 3.65 (dt, J = 9.0, 6.8 Hz, 1H), 4.22 (dd, J = 12.0, 2.9 Hz, 1H), 4.31 (dd, J = 12.0, 4.6 Hz, 1H), 4.69 (td, J = 4.8, 3.3 Hz, 1H), 6.77 (dd, J = 8.6, 2.9 Hz, 1H), 6.88 (d, J = 8.6 Hz, 1H), 7.09 (d, J = 3.9 Hz, 1H), 7.36 (d, J = 2.9 Hz, 1H). ^{13}C-NMR (125 MHz, $CDCl_3$): 167.9, 150.4, 141.8, 123.8, 121.5, 121.0, 117.2, 81.8, 73.8, 68.5, 31.5, 29.4, 25.6, 22.5, 13.9.

5-chloro-*N*-(3-(hexyloxy)-5-oxo-2,3,4,5-tetrahydrobenzo[*f*][1,4]oxazepin-7-yl)-1*H*-indole-2-carboxamide (8f)

5-Chloro-2-indolecarboxylic acid (61 mg, 0.31 mmol) was dissolved in DMF (1 mL), followed by sequential addition of HATU (118 mg, 0.31 mmol) in DMF (1 mL) and Triethylamine (110 mg, 1.09 mmol) in DMF (0.5 mL). The mixture was stirred at room temperature for 10 min, then treated with a solution of 7-amino-3-hexyloxy-3,4-dihydrobenzo[*f*][1,4]oxazepin-5(2*H*)-one (86 mg, 0.31 mmol) in DMF (1 mL). The reaction was heated to 45°C and stirred for 5 h. After cooling to room temperature, the mixture was poured into 30 mL of water and filtered. Purification by silica gel column chromatography (dichloromethane/methanol, 0%-2% gradient) afforded the desired product as a white solid (75 mg, 53%). HPLC analysis: 100%. m.p. 187-189℃.

ESI-MS *m/z*: 490.1 (M+Cl)$^-$.

^{1}H-NMR (500 MHz, d_6-DMSO): 0.77 (t, J = 6.2 Hz, 3H), 1.14 (s, 6H), 1.40 (s, 2H), 3.38 (dd, J = 14.6, 5.9 Hz, 1H), 3.51 (dd, J = 15.4, 6.5 Hz, 1H), 4.25 (d, J = 12.0 Hz, 1H), 4.41 (d, J = 10.3 Hz, 1H), 4.63 (s, 1H), 7.04 (d, J = 8.7 Hz, 1H), 7.22 (d, J = 8.5 Hz, 1H), 7.41 (s, 1H), 7.48 (d, J = 8.6 Hz, 1H), 7.76 (s, 1H), 7.97 (d, J = 8.3 Hz, 1H), 8.30 (s, 1H), 8.95 (d, J = 5.5 Hz, 1H), 10.33 (s, 1H), 11.88 (s, 1H). ^{13}C-NMR (125 MHz, d_6-DMSO): 167.2, 159.6, 153.5, 135.6, 133.6, 133.4, 128.6, 125.8, 124.8, 124.3, 123.9, 123.4, 121.2, 120.8, 114.4, 103.7, 80.0, 73.6, 67.1, 31.3, 29.3, 25.7, 22.5, 14.3.

3-(benzyloxy)-7-nitro-3,4-dihydrobenzo[*f*][1,4]oxazepin-5(2*H*)-one (6g)

7-Nitro-3-hydroxy-3,4-dihydrobenzo[*f*][1,4]oxazepin-5(2*H*)-one (0.16 g, 0.71 mmol) was dissolved in Benzyl acetate (8 mL), followed by addition of P-toluenesulfonic acid (15 mg, 0.09 mmol). The mixture was heated to 50°C and stirred for 18 h. After cooling to room temperature, the solvent was concentrated under reduced pressure. Purification by silica gel column chromatography (petroleum ether/ethyl acetate, 15%-50% gradient) afforded the desired product as a white solid (135 mg, 60%).HPLC analysis: 100%. m.p. 171-173℃.

ESI-MS *m/z*: 337.2 (M+Na)$^+$.

^{1}H-NMR (500 MHz, $CDCl_3$): 4.35 (d, *J* = 12.4 Hz, 1H), 4.63-4.70 (m, 2H), 4.80 (dd, *J* = 14.9, 8.8 Hz, 2H), 7.19 (d, *J* = 9.0 Hz, 1H), 7.32-7.39 (m, 5H), 8.26 (dd, *J* = 9.0, 2.7 Hz, 1H), 8.68 (s, 1H), 9.21 (d, *J* = 2.6 Hz, 1H). ^{13}C-NMR (125 MHz, $CDCl_3$): 166.4, 162.0, 142.3, 136.2, 130.5, 128.8, 128.4, 128.3, 128.2, 121.9, 120.0, 77.6, 72.5, 69.4.

7-amino-3-(benzyloxy)-3,4-dihydrobenzo[*f*][1,4]oxazepin-5(2*H*)-one (7g)

3-Benzyloxy-7-nitro-3,4-dihydrobenzo[*f*][1,4]oxazepin-5(2*H*)-one (105 mg, 0.33 mmol) was dissolved in THF (15 mL), followed by addition of Ammonium formate (300 mg, 4.76 mmol) and Palladium on Carbon (15 mg, 0.14 mmol). The mixture was heated to 50°C and stirred for 4 h under hydrogen atmosphere. After cooling to room temperature, the reaction mixture was filtered through Celite and concentrated under reduced pressure. Purification by silica gel column chromatography (dichloromethane/methanol, 0%-2% gradient) afforded the product as a colorless viscous liquid (80 mg, 84%). HPLC analysis: 95%.

ESI-MS *m/z*: 285.2 $(M+H)^+$.

^{1}H-NMR (500 MHz, d_6-DMSO): 4.17-4.23 (m, 2H), 4.42 (d, *J* = 12.0 Hz, 1H), 4.56 (d, *J* = 12.0 Hz, 1H), 4.68-4.69 (m, 1H), 4.91 (s, 2H), 6.66 (dd, *J* = 8.5, 2.7 Hz, 1H), 6.74 (d, *J* = 8.5 Hz, 1H), 7.08 (d, *J* = 2.6 Hz, 1H), 7.20 (d, *J* = 7.2 Hz, 2H), 7.27 (dq, *J* = 14.3, 7.2 Hz, 3H), 8.82 (d, *J* = 5.5 Hz, 1H). ^{13}C-NMR (125 MHz, d_6-DMSO): 168.4, 148.3, 144.4, 138.3, 128.6, 128.0, 127.9, 125.5, 121.0, 119.5, 115.7, 80.5 , 74.5, 68.8.

***N*-(3-(benzyloxy)-5-oxo-2,3,4,5-tetrahydrobenzo[*f*][1,4]oxazepin-7-yl)-5-chloro-1*H*-indole-2-carboxamide (8g)**

5-Chloroindole-2-carboxylic acid (59 mg, 0.30 mmol) was dissolved in DMF (1 mL), followed by sequential addition of HATU (114 mg, 0.30 mmol) in DMF (1 mL) and Triethylamine (0.15 mL, 1.05 mmol) in DMF (0.5 mL). The mixture was stirred at room temperature for 10 min. Then, a solution of 7-amino-3-benzyloxy-3,4-dihydrobenzo[*f*][1,4]oxazepin-5(2*H*)-one (85 mg, 0.30 mmol) in DMF (1 mL) was added, and the reaction was heated to 45°C for 5 h. After cooling to room temperature, the mixture was diluted with water (30 mL) and filtered. Purification by silica gel column chromatography (dichloromethane/methanol, 0%-2% gradient) afforded the product as a white solid (63 mg, 46%). HPLC analysis: 100%. m.p. 253-255°C.

ESI-MS *m/z*: 497.4 $(M+Cl)^-$.

^{1}H-NMR (500 MHz, d_6-DMSO): 4.29 (dd, *J* = 12.3, 1.4 Hz, 1H), 4.47-4.52 (m, 2H), 4.63 (d, *J* = 12.0

Hz, 1H), 4.77-4.78 (m, 1H), 7.07 (d, *J* = 8.8 Hz, 1H), 7.24 (dt, *J* = 6.5, 3.9 Hz, 4H), 7.28-7.31 (m, 2H), 7.43 (s, 1H), 7.49 (d, *J* = 8.7 Hz, 1H), 7.77 (d, *J* = 1.3 Hz, 1H), 7.98 (dd, *J* = 8.8, 2.6 Hz, 1H), 8.36 (d, *J* = 2.6 Hz, 1H), 9.11 (d, *J* = 6.0 Hz, 1H), 10.36 (s, 1H), 11.90 (s, 1H). ^{13}C-NMR (125 MHz, d_6-DMSO): 167.1, 159.6, 153.7, 138.2, 135.6, 133.6, 133.4, 128.7, 128.6, 128.0, 127.9, 126.1, 124.9, 124.3, 124.1, 123.1, 121.2, 120.8, 114.4, 103.8, 79.6, 73.3, 68.8.

3-(isopentyloxy)-7-nitro-3,4-dihydrobenzo[*f*][1,4]oxazepin-5(2*H*)-one (6h)

7-Nitro-3-hydroxy-3,4-dihydrobenzo[*f*][1,4]oxazepin-5(2*H*)-one (0.20 g, 0.89 mmol) was dissolved in Isoamyl alcohol (8 mL), followed by addition of P-toluenesulfonic acid (20 mg, 0.12 mmol). The mixture was heated to 50°C and stirred for 15 h. After cooling to room temperature, the solvent was concentrated under reduced pressure. Purification by silica gel column chromatography (petroleum ether/ethyl acetate, 15%-45% gradient) afforded the product as a white solid (0.18 g, 68%). HPLC analysis: 100%. m.p. 126-128℃.

ESI-MS *m/z*: 295.2 (*M*+H)$^+$.

^{1}H-NMR (500 MHz, $CDCl_3$): 0.82 (dd, *J* = 9.3, 6.6 Hz, 6H), 1.45 (tt, *J* = 13.7, 6.7 Hz, 2H), 1.55 (dt, *J* = 13.4, 6.7 Hz, 1H), 3.56 (dt, *J* = 9.2, 6.6 Hz, 1H), 3.74 (dt, *J* = 9.2, 6.9 Hz, 1H), 4.37 (d, *J* = 12.2 Hz, 1H), 4.70 (dd, *J* = 12.3, 4.3 Hz, 1H), 4.80 (t, *J* = 3.6 Hz, 1H), 7.16 (d, *J* = 9.1 Hz, 1H), 8.25 (dd, *J* = 9.1, 2.9 Hz, 1H), 8.42 (d, *J* = 4.9 Hz, 1H), 9.15 (d, *J* = 2.9 Hz, 1H). ^{13}C-NMR (125 MHz, $CDCl_3$): 166.1, 162.0, 142.3, 130.3 , 128.1, 121.8, 120.3, 79.6, 72.6, 66.8, 38.0, 24.9, 22.5, 22.2.

7-amino-3-(isopentyloxy)-3,4-dihydrobenzo[*f*][1,4]oxazepin-5(2*H*)-one (7h)

3-Isopentoxy-7-nitro-3,4-dihydrobenzo[*f*][1,4]oxazepin-5(2*H*)-one (150 mg, 0.51 mmol) was dissolved in Tetrahydrofuran (20 mL), followed by addition of Ammonium formate (400 mg, 6.35 mmol) and Palladium on Carbon (15 mg, 0.14 mmol). The mixture was heated to 50°C and stirred for 4 h. After cooling to room temperature, the reaction mixture was filtered through Celite, concentrated under reduced pressure, and purified by silica gel column chromatography (dichloromethane/methanol, 0%-2% gradient) to afford the product as a colorless viscous oil (115 mg, 85%). HPLC analysis: 92.6%.

ESI-MS *m/z*: 265.2 (*M*+H)$^+$.

^{1}H-NMR (500 MHz, d_6-DMSO): 0.78 (d, *J* = 6.7 Hz, 6H), 1.20-1.32 (m, 2H), 1.42-1.51 (m, 1H), 3.30-3.35 (m, 1H), 3.52 (dt, *J* = 9.5, 6.9 Hz, 1H), 4.14 (d, *J* = 3.2 Hz, 2H), 4.89 (s, 2H), 4.52-4.56 (m, 1H), 6.65 (dd, *J* = 8.5, 2.8 Hz, 1H), 6.72 (dd, *J* = 8.5, 2.3 Hz, 1H), 7.05 (d, *J* = 2.8 Hz, 1H), 8.67 (d, *J* = 5.9 Hz, 1H). ^{13}C-NMR (125 MHz, d_6-DMSO): 168.3, 148.2, 144.3, 125.6, 121.0, 119.4, 115.7, 80.9,

74.6, 65.6, 38.3, 24.8, 23.0, 22.6.

5-chloro-*N*-(3-(isopentyloxy)-5-oxo-2,3,4,5-tetrahydrobenzo[*f*][1,4]oxazepin-7-yl)-1*H*-indole-2-carboxamide (8h)

5-Chloroindole-2-carboxylic acid (78 mg, 0.40 mmol) was dissolved in N,N-dimethylformamide (1 mL), followed by sequential addition of HATU (152 mg, 0.40 mmol) in DMF (1 mL) and Triethylamine (0.19 mL, 1.40 mmol) in DMF (0.5 mL). The mixture was stirred at room temperature for 10 min, then treated with 7-amino-3-isopentoxy-3,4-dihydrobenzo[*f*][1,4]oxazepin-5(2*H*)-one (105 mg, 0.40 mmol) in DMF (1 mL). The reaction was heated to 45°C and stirred for 5 h. After cooling, the mixture was diluted with water (30 mL), filtered, and purified by silica gel column chromatography (dichloromethane/methanol, 0%-2% gradient) to afford the product as a white solid (81 mg, 46%). HPLC analysis: 100%. m.p. 95-97℃.

ESI-MS *m/z*: 477.9 $(M+Cl)^-$.

^{1}H-NMR (500 MHz, d_6-DMSO): 0.78 (dd, *J* = 6.6, 1.7 Hz, 6H), 1.21-1.37 (m, 2H), 1.45-1.53 (m, 1H), 3.41 (dd, *J* = 15.9, 6.4 Hz, 1H), 3.58 (dd, *J* = 16.4, 7.0 Hz, 1H), 4.24 (d, *J* = 12.1 Hz, 1H), 4.42 (dd, *J* = 12.3, 3.2 Hz, 1H), 4.64 (d, *J* = 2.2 Hz, 1H), 7.04 (d, *J* = 8.8 Hz, 1H), 7.22 (dd, *J* = 8.7, 1.7 Hz, 1H), 7.42 (s, 1H), 7.49 (d, *J* = 8.7 Hz, 1H), 7.76 (s, 1H), 7.96 (dd, *J* = 8.8, 2.3 Hz, 1H), 8.29-8.34 (m, 1H), 8.95 (s, 1H), 10.33 (s, 1H), 11.87 (s, 1H). ^{13}C-NMR (125 MHz, d_6-DMSO): 167.1, 159.6, 153.6, 135.6, 133.5, 133.4, 128.6, 125.9, 124.9, 124.3, 124.0, 123.1, 121.2, 120.8, 114.4, 103.7, 80.0, 73.4, 65.5, 38.2, 24.9, 22.9, 22.6.

3-(2-methoxyethoxy)-7-nitro-3,4-dihydrobenzo[*f*][1,4]oxazepin-5(2*H*)-one (6i)

7-Nitro-3-hydroxy-3,4-dihydrobenzo[*f*][1,4]oxazepin-5(2*H*)-one (0.39 g, 1.74 mmol) was dissolved in Ethylene glycol methyl ether acetate (6 mL). p-Toluenesulfonic acid (30 mg, 0.17 mmol) was added under stirring, and the mixture was heated to 50°C with stirring for 15 h. After cooling to room temperature, the reaction mixture was concentrated under reduced pressure. Purification by silica gel column chromatography (petroleum ether/ethyl acetate, 15–45% gradient elution) afforded the desired product as a white solid (0.29 g, 59% yield). HPLC analysis: 94%. m.p. 115-117℃.

ESI-MS *m/z*: 305.1 $(M+Na)^+$.

^{1}H-NMR (500 MHz, $CDCl_3$): 3.37 (s, 3H), 3.55-3.56 (m, 2H), 3.78-3.87 (m, 2H), 4.36 (d, *J* = 12.4 Hz, 1H), 4.70 (dd, *J* = 12.4, 4.5 Hz, 1H), 4.95 (t, *J* = 5.0 Hz, 1H), 7.16 (d, *J* = 9.0 Hz, 1H), 8.12 (d, *J* = 4.3 Hz, 1H), 8.24 (dd, *J* = 9.0, 2.9 Hz, 1H), 9.18 (d, *J* = 2.9 Hz, 1H). ^{13}C-NMR (125 MHz, $CDCl_3$):

165.3, 162.1, 142.4, 130.5, 128.1, 121.7, 120.4, 80.3, 72.41, 72.38, 67.7, 59.0.

7-amino-3-(2-methoxyethoxy)-3,4-dihydrobenzo[*f*][1,4]oxazepin-5(2*H*)-one (7i)

3-(2-Methoxyethoxy)-7-nitro-3,4-dihydrobenzo[*f*][1,4]oxazepin-5(2*H*)-one (0.16 g, 0.57 mmol) was dissolved in Tetrahydrofuran (20 mL). Ammonium formate (0.40 g, 6.35 mmol) and Palladium on Carbon (20 mg, 0.19 mmol) were added under stirring, and the mixture was heated to 50°C with stirring for 4 h. After cooling to room temperature, the reaction mixture was filtered through Celite and concentrated under reduced pressure. Purification by silica gel column chromatography (dichloromethane/methanol, 0–2% gradient elution) afforded the desired product as a colorless viscous oil (0.12 g, 84% yield). HPLC analysis: 92.6%.

ESI-MS *m/z*: 275.1 $(M+Na)^+$.

^{1}H-NMR (500 MHz, d_6-DMSO): 3.17 (s, 3H), 3.42-3.34 (m, 2H), 3.53-3.45 (m, 1H), 3.60 (ddd, *J* = 10.6, 5.5, 4.1 Hz, 1H), 4.15 (qd, *J* = 12.3, 3.2 Hz, 2H), 4.61 (dt, *J* = 6.1, 3.2 Hz, 1H), 4.89 (s, 2H), 6.66 (dd, *J* = 8.5, 2.8 Hz, 1H), 6.73 (d, *J* = 8.5 Hz, 1H), 7.09 (d, *J* = 2.7 Hz, 1H), 8.67 (d, *J* = 5.5 Hz, 1H).

^{13}C-NMR (125 MHz, d_6-DMSO): 168.0, 148.4, 144.3, 125.1, 121.0, 119.6, 115.8, 81.3, 74.2, 71.3, 66.7, 58.4.

5-chloro-*N*-[3-(2-methoxyethoxy)-5-oxo-2,3,4,5-tetrahydrobenzo[*f*][1,4]oxazepin-7-yl]-1*H*-indole-2-carboxamide (8i)

5-Chloro-1H-indole-2-carboxylic acid (78 mg, 0.40 mmol) was dissolved in N,N-dimethylformamide (1 mL). To this solution was added a solution of HATU (150 mg, 0.40 mmol) in N,N-dimethylformamide (1 mL) followed by Triethylamine (0.15 mL, 1.05 mmol) in N,N-dimethylformamide (0.5 mL). The mixture was stirred at room temperature for 10 min. A solution of 7-amino-3-(2-methoxyethoxy)-3,4-dihydrobenzo[*f*][1,4]oxazepin-5(2*H*)-one (100 mg, 0.40 mmol) in N,N-dimethylformamide (1 mL) was added, and the reaction was heated to 45°C with stirring for 5 h. After cooling to room temperature, the reaction mixture was diluted with water (30 mL) and filtered. Purification by silica gel column chromatography (dichloromethane/methanol, 0–2% gradient elution) afforded the desired product as a white solid (100 mg, 59% yield). HPLC analysis: 99%. m.p. 206-208℃.

ESI-MS *m/z*: 465.9 $(M+Cl)^-$.

^{1}H-NMR (500 MHz, d_6-DMSO): 3.18 (s, 3H), 3.39 (tt, *J* = 10.8, 5.6 Hz, 2H), 3.54-3.58 (m, 1H), 3.64-3.69 (m, 1H), 4.23 (d, *J* = 12.4 Hz, 1H), 4.44 (dd, *J* = 12.4, 3.6 Hz, 1H), 4.69-4.70 (m, 1H), 7.05

(d, J = 8.8 Hz, 1H), 7.22 (dd, J = 8.7, 1.9 Hz, 1H), 7.42 (s, 1H), 7.48 (d, J = 8.7 Hz, 1H), 7.76 (s, 1H), 7.98 (dd, J = 8.8, 2.7 Hz, 1H), 8.34 (d, J = 2.7 Hz, 1H), 8.97 (d, J = 5.8 Hz, 1H), 10.35 (s, 1H), 11.89 (s, 1H). ^{13}C-NMR (125 MHz, d_6-DMSO): 166.3, 159.1, 153.2, 135.1, 133.0, 132.9, 128.0, 125.5, 124.3, 123.7, 122.1, 120.7, 120.3, 113.9, 103.2, 79.8, 72.4, 70.8, 66.2, 57.9.
