# Supplementary material for: Synthesis and Activity Evaluation of Novel Benzoxazepinone Derivatives as Potential Inhibitors of Glycogen Phosphorylase
Source: Molecules. 2025 Oct 31;30(21):4249. doi: 10.3390/molecules30214249 (PMC12608647; doi:10.3390/molecules30214249)
Supplement: Supplementary file 1 [file molecules-30-04249-s001.zip › The mass spectrum of compound 8a-i.pdf]

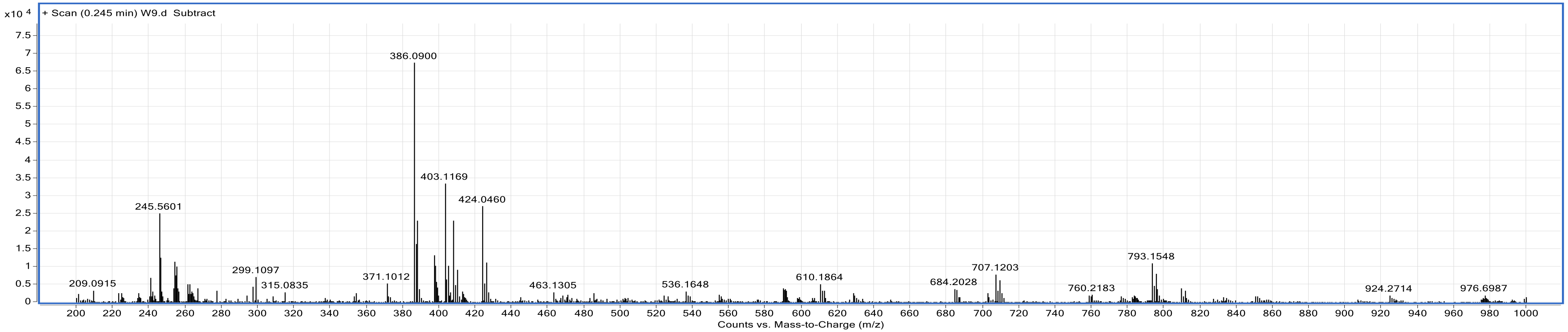

The mass spectrum of compound **8a**

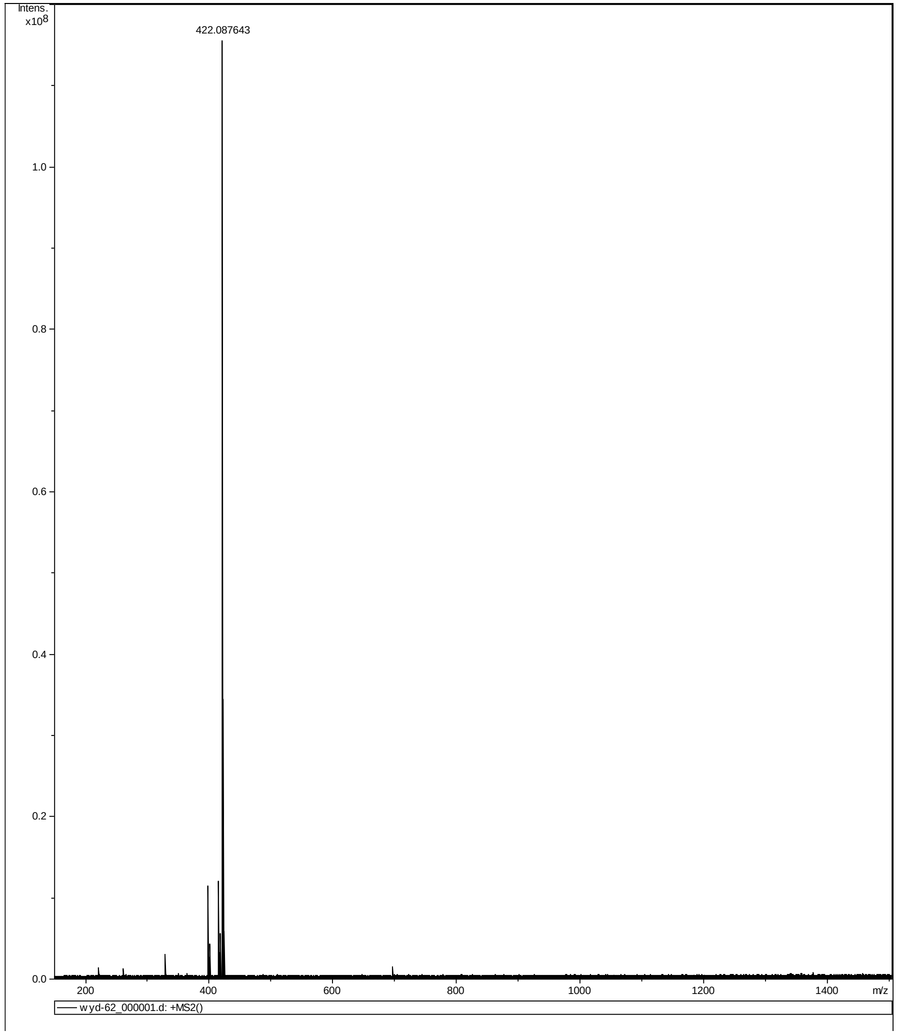

The mass spectrum of compound **8b**

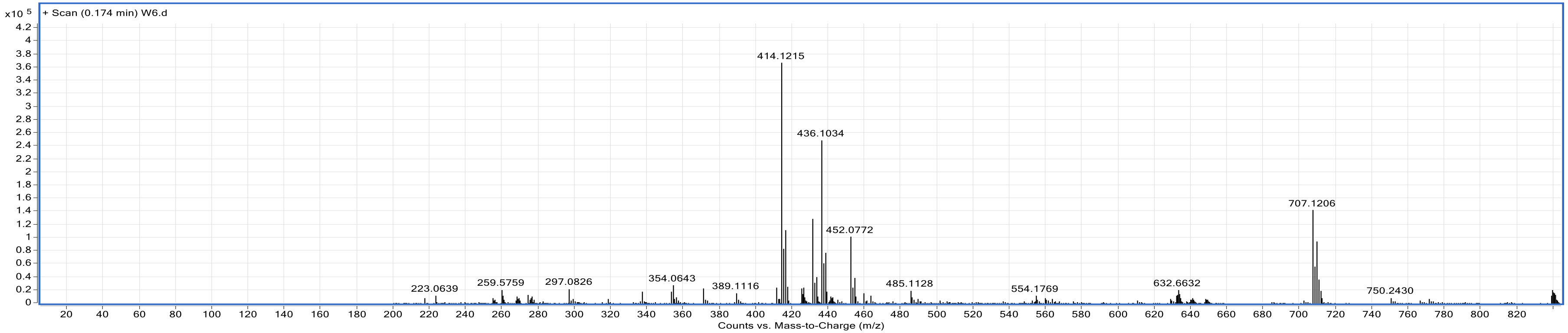

The mass spectrum of compound **8c**

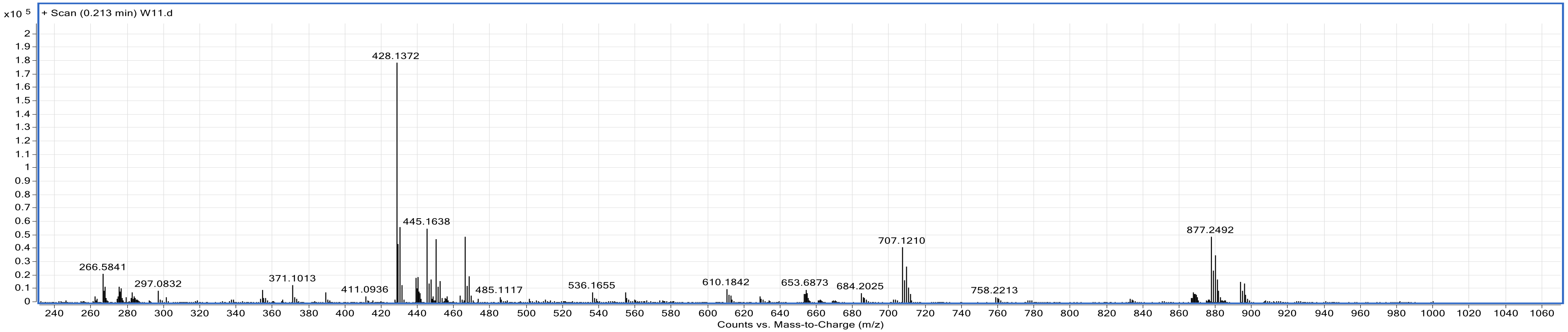

The mass spectrum of compound **8d**

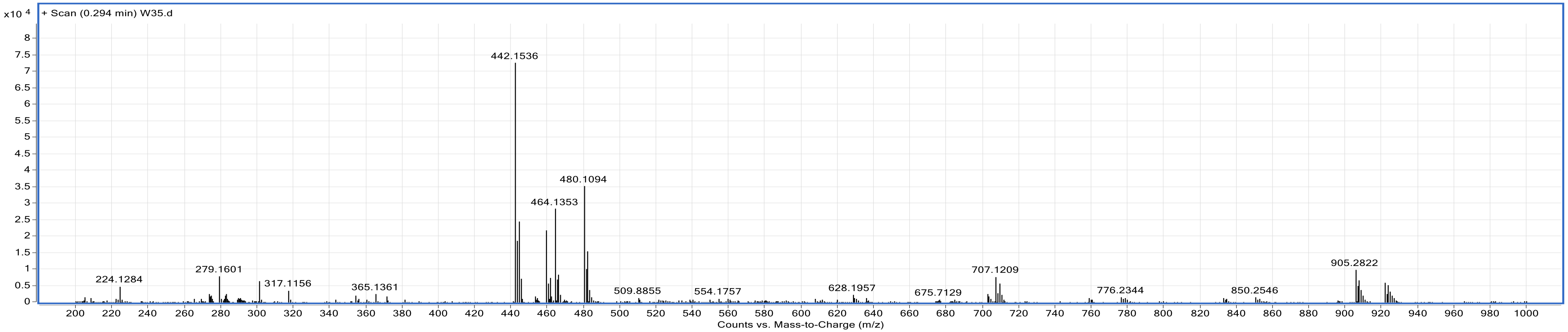

The mass spectrum of compound **8e**

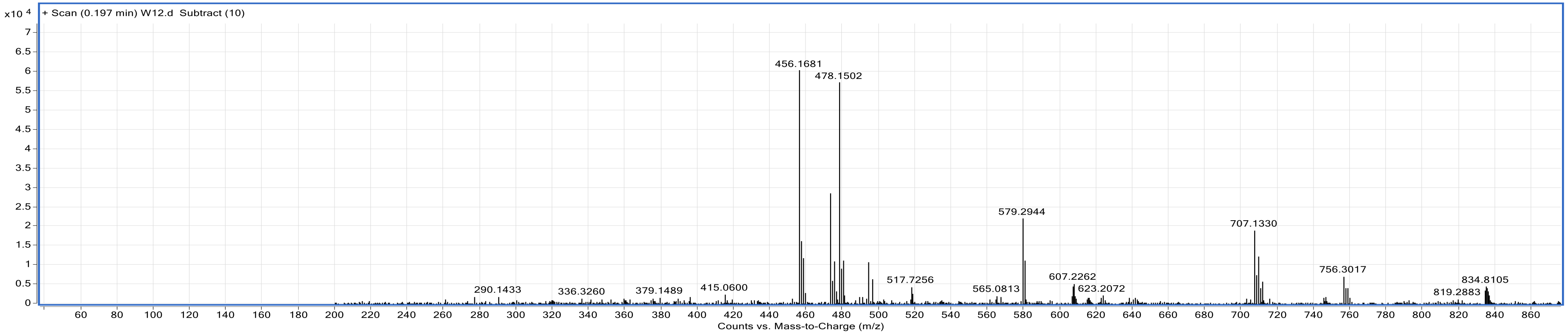

The mass spectrum of compound **8f**

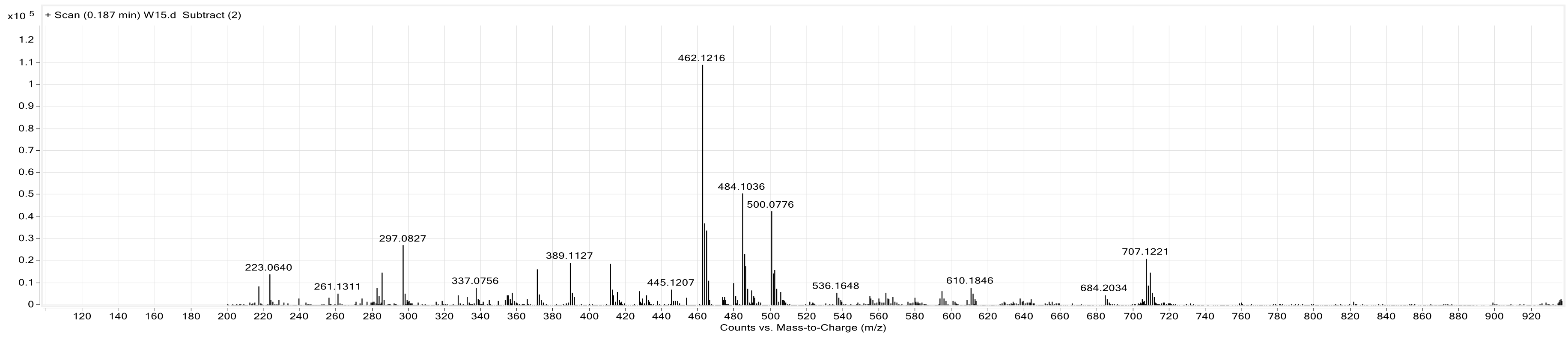

The mass spectrum of compound **8g**

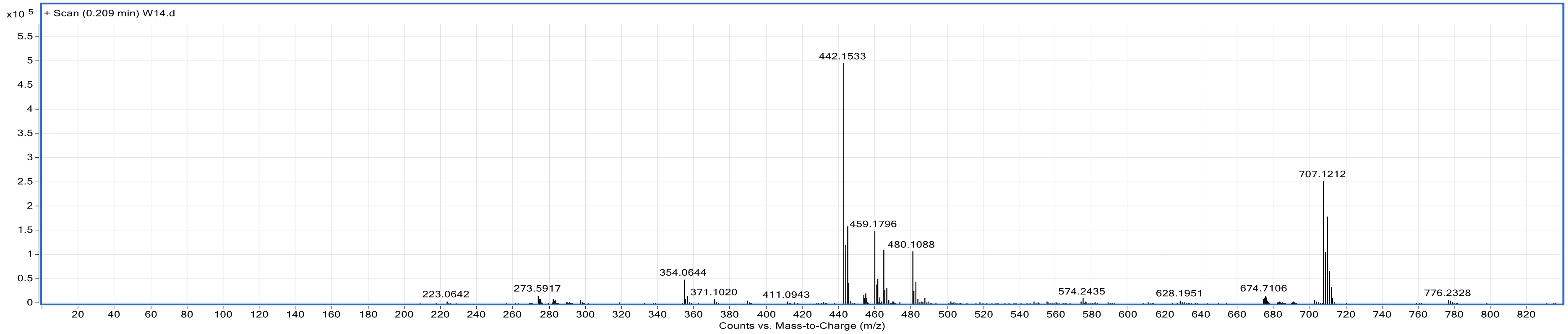

The mass spectrum of compound **8h**

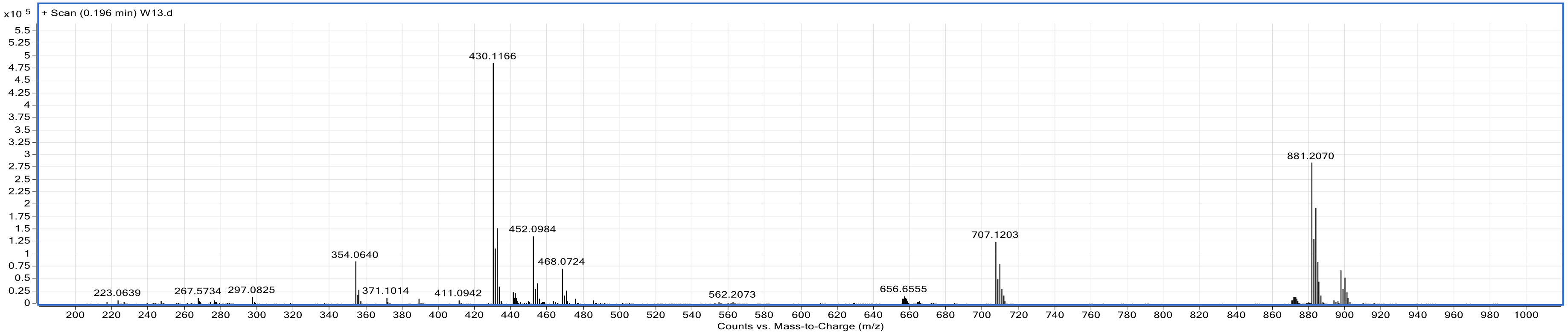

The mass spectrum of compound **8i**
